# Non-Alcoholic Beer Influences Glucose and Lipid Metabolism and Changes Body Composition in Healthy, Young, Male Adults

**DOI:** 10.3390/nu17101625

**Published:** 2025-05-09

**Authors:** Henriette Kreimeyer, Svenja Sydor, Lara Buchholz, Cagatay Toskal, Mustafa Özcürümez, Bernd Schnabl, Wing-Kin Syn, Jan-Peter Sowa, Paul Manka, Ali Canbay

**Affiliations:** 1 Department of Medicine, Knappschaft Kliniken University Hospital Bochum, Ruhr-University Bochum, 44801 Bochum, Germany; jkreimeyer@health.ucsd.edu (H.K.); svenja.sydor@rub.de (S.S.); lara.kaiser@rub.de (L.B.); cagatay.toskal@rub.de (C.T.); mustafa.oezcueruemez@knappschaft-kliniken.de (M.Ö.); jan.sowa@rub.de (J.-P.S.); ali.canbay@rub.de (A.C.); 2 Department of Medicine, University of California San Diego, San Diego, CA 92093, USA; beschnabl@health.ucsd.edu; 3 Department of Medicine, VA San Diego Healthcare System, San Diego, CA 92161, USA; 4 Division of Gastroenterology and Hepatology, Saint Louis University, St. Louis, MO 63103, USA; wingkin.syn@health.slu.edu; 5 Department of Physiology, Faculty of Medicine and Nursing, University of Basque Country UPV/EHU, 48940 Leioa, Spain

**Keywords:** liver, microbiome, non-alcoholic beer, glucose metabolism, fat metabolism

## Abstract

**Background and Aims:** Non-alcoholic beers (NABs) are gaining popularity as alternatives to alcoholic beverages, yet their metabolic and health effects compared to no consumption of these drinks remain unclear. **Material and Methods:** The investigator-blinded, single-center, randomized study compares the effects on the metabolism, health, and gut microbiome of the daily consumption of different NABs—pilsener, mixed beer, and wheat beer—on glucose and fat metabolism, body composition, and liver function in 44 healthy young men. The participants consumed 660 mL of one of these beers or water daily for 4 weeks. We measured indicators of glucose and lipid metabolism, liver enzymes, body composition, and the composition of the gut microbiota. **Results:** The findings revealed that mixed beer increased fasting glucose and triglycerides, and wheat beer increased insulin, C-peptide, and triglycerides. The intake of pilsener and water decreased cholesterol and LDL levels without significantly affecting glucose metabolism. Biomarkers of liver damage such as M30 lowered in water and pilsener, while ALT and AST lowered in mixed beer. The pattern of the gut microbiota also changed, as pilsener lowered *Firmicutes* and increased *Actinobacteria*. **Conclusions:** In summary, consumption of NABs, especially mixed and wheat beers, exerts an unfavorable metabolic impact on glucose and fat, while pilsener and water are more favorable from a metabolic perspective. We concluded that the metabolic alterations seen are probably due to the caloric and sugar content in NABs, rather than polyphenols. The chronic effects of NABs on health should be evaluated in future studies.

## 1. Introduction

Non-alcoholic beer (NAB) is increasingly consumed as an alternative to alcoholic drinks, which is boosting the market share of NAB drinks [1]. Regular alcohol consumption harms the liver, pancreas, peripheral and central nervous systems, and cardiovascular system, as well as promoting the development of metabolic syndrome and cancers [2,3,4,5,6]. The WHO’s recommendation to avoid the consumption of alcoholic beverages prompts the population to reflect on their alcohol consumption, and many aim to reduce it [7,8]. There are numerous breweries and beverage manufacturers who are responding to this customer demand and serving the market with NAB alternatives. Classic NAB brewed in the Pilsener style is popular, as are non-alcoholic wheat and mixed beer drinks with added lemon or orange soda. Several studies have compared the effect of NAB consumption to moderate alcoholic beer consumption [9,10,11,12,13]. However, they lack comparison to NAB or alcoholic beer (AB) consumption and the influence of different styles of NAB.

The benefits or risks of consuming NAB as an alternative to AB are not yet known. In our study, we investigate the effects of the regular consumption of these drinks on the liver, glucose, and fat metabolism, as well as body composition.

## 2. Material and Methods

### 2.1. Patient Recruitment, Ethical Statement, and Sample Collection

Patients were prospectively recruited in the Department of Medicine at the Knappschaft Kliniken University Hospital Bochum from March 2022 to November 2023 and were examined before NAB consumption and after one month. The study was approved by the local Ethics committee of the Ruhr University Bochum (RUB) (Institutional Review Board; reference number: 20-7019) and was registered at the German Clinical Trial Register (DRKS/No. DRKS00027109). All subjects provided informed written consent, and the study protocol followed the ethical guidelines of the Declaration of Helsinki.

The study size was calculated according to similar studies that measured the influence of moderate beer consumption on glucose metabolism, cardiovascular disease, and microbiota [9,14]. We have defined the onset of fatty degeneration of the liver by the increase in serum triglycerides and transaminases AST/ALT above the limit values as the primary outcome and to calculate Cohen’s d in order to define an effect size for the study. Based on the results of these studies, we calculated the effect size and computed the required sample size using G*Power, Version 3.1. For an effect size of 0.6 and a power of 0.8, we calculated a sample size of 24 per group to receive significant results with a Wilcoxon matched pairs signed rank test (*p* < 0.05), whereby the expected recruitment figure could not be achieved. One subject was excluded from the water control group because he did not attend the scheduled visits to obtain measurements and samples. The analysis follows the per protocol approach.

We included adult male subjects (18–30 years, non-smoker, and BMI < 30). The exclusion criteria were general abstinence from alcohol and addictions, especially alcohol addiction, and this was determined in advance using the AUDIT (Alcohol Use Disorders Identification Test) questionnaire. Any chronic inflammatory bowel disease or active malignant disease were defined as hard exclusion criteria. Before the intervention, a wash-out period was implemented, where the subjects did not drink any alcohol for 4 weeks and did not change eating or exercise habits. To assess the effect of NAB on human health, the subjects were asked to consume 660 mL (2 bottles á 330 mL/bottle) of either pilsener, mixed beer, or wheat beer per day for 4 weeks. The quantity of 660 mL corresponds to 2 small standard bottles of beer/NAB, and thus corresponds to a usual daily amount of beer or NAB consumed in Germany. A control group of men consumed water. The study participants were randomized into one of the four groups (principle of coincidence) using a randomization list previously created with the “randomizr package” in R (version 4.4.2) before the study. Body composition was assessed using bioelectrical impedance analysis (BIA) and we collected fecal and serum samples at both baseline (week 0) and after the 4-week intervention (week 4). The serum samples were collected in a fasted state and stored in aliquots at −80 °C until analysis. The standard laboratory parameters were analyzed in the central laboratory of our hospital using the cobas6000 Clinical chemistry analyzer system (Roche Diagnostics, Penzberg, Germany). Adiponectin and M30 in serum were determined via ELISA (IBL International, Hamburg, Germany). The baseline characteristics are shown in Table 1.

### 2.2. Assessment of Liver Steatosis via Transient Elastography

We combined transient elastography measurement (TEM) via Fibroscan^®^ with the controlled attenuation parameter (CAP) to measure the hepatic fat accumulation. The body fat mass was determined via bioelectrical impedance analysis (BIA) using the BodyExplorer (Juwell medical GmbH, Rheine, Germany).

### 2.3. Collection of Fecal Samples and Preparation for Sequencing

Fecal samples were collected from every subject in sterile tubes and stored at −80 °C until bacterial DNA isolation. Bacterial DNA was isolated using the QIamp-DNA isolation kit following the manufacturer’s instructions (Qiagen, Hilden, Germany), including a mechanical lysis step using dry bead tubes (MoBio Laboratories Inc., Carlsbad, CA, USA) and the Fast Prep™-24 instrument (MP Biomedicas, Solon, OH, USA) at 6.0 m/s for 45 sec (two times). PCR amplification of bacterial 16sRNA (V3-V4 region) was performed using the 341F (5′-CTACGGGNGGCWGCAG-3′)/806R (5′-GGACTACNNGGGTATCT AAT-3′) Primers (Eurofins Genomics Europe, Ebersberg, Germany). The amplification of targeted regions was performed using specific primers connecting with barcodes. The PCR products of a proper size were selected through 2% agarose gel electrophoresis. The same amount of PCR products from each sample was pooled, end-repaired, A-tailed, and further ligated with Illumina adapters. Libraries were sequenced on a paired-end Illumina platform. The library was checked with Qubit and real-time PCR for quantification, while a Bioanalyzer was used for size distribution detection. Quantified libraries were pooled and sequenced on Illumina platforms according to the effective library concentration and data amount required. The amplicon was sequenced on an Illumina paired-end platform to generate 250 bp paired-end raw reads (Raw PE) and then merged and pre-treated to obtain Clean Tags. The chimeric sequences in Clean Tags were detected and removed to obtain the effective tags, which can be used for subsequent analysis. Library preparation and sequencing were carried out in Novogene (Novogene Sequencing, Amsterdam, The Netherlands).

### 2.4. Sequencing Data Analysis

Noise reduction was performed using the DADA2 method, and as result, a unique table containing all samples with the feature sequences and abundances was generated. A pre-trained Naïve Bayesian classification was used for the species annotation of each amplicon sequence variant by applying the QIIME2’s classify-sklearn algorithm [15,16]. The annotation database Silva 138.1 was used for the project. Relative species abundance tables at the level of kingdom, Phyla, Class, Order, Family, Genus, and Species were obtained and used for further downstream analyses.

### 2.5. Statistical Analysis

Statistical analysis was performed using R (version 4.4.2). Repeated measurements ANOVA was calculated using the *afex* (version 1.4.1.) and *emeans* (version 1.11.) package. Alpha and beta-Diversity, microbial diversity based on the Shannon, Chao 1, and inverse Simpson indices was calculated using the *phyloseq* (version 1.52.0.) and *microbiome* (version 1.30.) package [15,17]. Significance for beta-Diversity was calculated using permutational multivariate analysis of variance (Permanova) [16]. The comparison of more than two groups was performed using the Kruskal–Wallis test and the comparison of two groups was performed using the Wilcoxon signed rank test on paired samples if eligible. Figures were designed using *ggplot* and *ggpubr*. If not stated otherwise, all data are presented as means ± SEM, and statistical significance was assumed at *p* ≤ 0.05.

## 3. Results

### 3.1. Impact of Mixed Beer and Wheat Beer on Glucose and Fat Metabolism

At baseline, body characteristics did not show any statistically significant differences. Bodyfat, BMI, and waist and hip circumferences were lower in the mixed beer group compared to the other groups, while triglycerides were lower in the wheat beer group (Table 1). Comparison of calorie, sugar, alcohol, and polyphenol content of the included non-alcoholic beverages is shown in Table 2.

**Figure 1:**
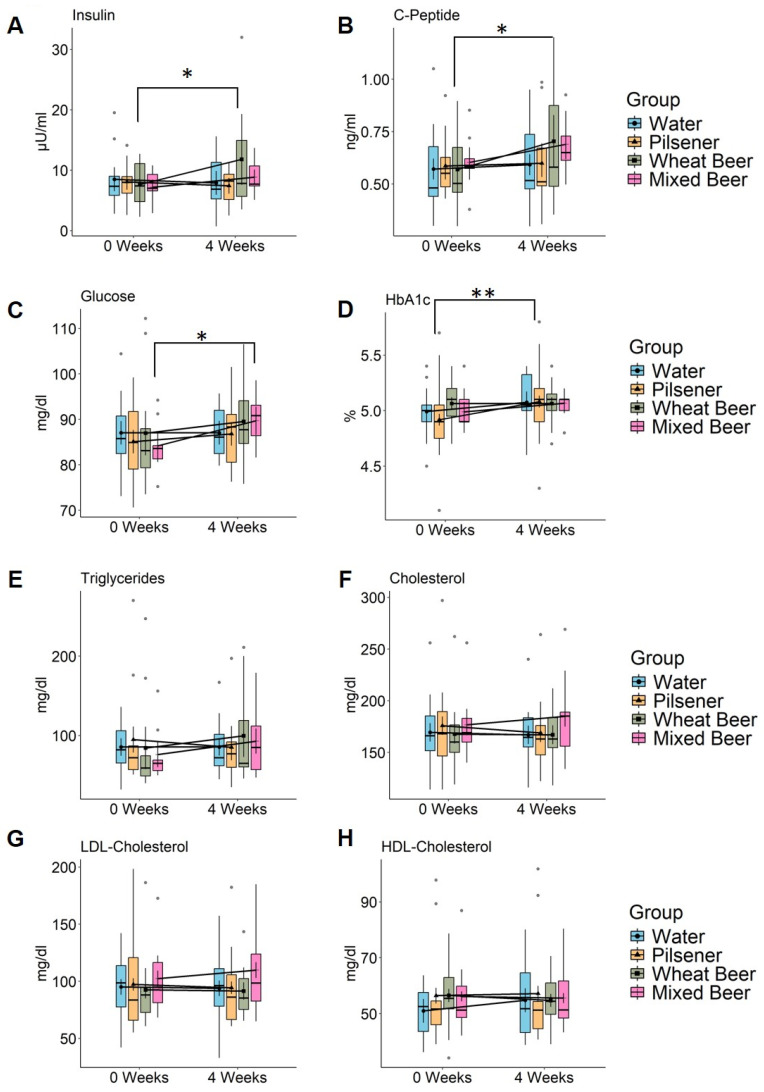
Non-alcoholic beer consumption alters glucose and lipid metabolism in healthy, young men. Markers of glucose and fat metabolism were measured in healthy young men at baseline and after 4 weeks consumption of 660 mL daily of either pilsener (PI; n = 11), wheat beer (WB; n = 11), or mixed beer (MB; n = 10). Water consumption (WA; n = 12) was included as a control group. Estimated marginal means and error bars (95% confidence interval) are plotted in in the foreground and raw data are plotted in the background. Significance was calculated using two-way repeated measures ANOVA. Significant difference between time points using pairwise comparison of estimated marginal means is marked in the plot (* *p* < 0.05; ** *p* < 0.01). (**A**) Insulin (WA: *p* = 0.58, PI: *p* = 0.36, WB: *p* = 0.014, MB: *p* = 0.36), (**B**) C-peptide (WA: *p* = 0.68, PI: *p* = 0.81, WB: *p* = 0.134, MB: *p* = 0.14), (**C**) fasting glucose (WA: *p* = 0.99, PI: *p* = 0.44, WB: *p* = 0.24, MB: *p* = 0.027), (**D**) HbA1c (WA: *p* = 0.08, PI: *p* = 0.0016, WB: *p* = 1, MB: *p* = 0.15), (**E**) triglycerides (WA: *p* = 0.52, PI: *p* = 0.23, WB: *p* = 0.32, MB: *p* = 0.32), (**F**) cholesterol (WA: *p* = 0.65, PI: *p* = 0.24, WB: *p* = 0.91, MB: *p* = 0.2), (**G**) LDL cholesterol (WA: *p* = 0.77, PI: *p* = 0.49, WB: *p* = 0.8, MB: *p* = 0.12), and (**H**) HDL cholesterol (WA: *p* = 0.05, PI: *p* = 0.7, WB: *p* = 0.37, MB: *p* = 0.78). LDL = low-density lipoprotein and HDL = high-density lipoprotein.

We measured insulin, C-peptide, fasting glucose, and HbA1c in the serum as indicators for glucose metabolism and total cholesterol, low-density lipoprotein (LDL) cholesterol, high-density lipoprotein (HDL) cholesterol, and triglycerides in the serum for fat metabolism. Insulin, C-peptide, and fasting glucose increased in the wheat beer and mixed beer group (Figure 1A–C, Supplementary Table S1), while no changes were observed in the pilsener and water groups. HbA1c increased by 0.16 percent points in the pilsener group (*p* = 0.0016) and by 0.08 percent point in the mixed beer group (*p* = 0.15) and water group (*p* = 0.08), but remained unchanged in the wheat beer group (*p* = 1.0) (Figure 1D, Supplementary Table S1).

The triglyceride (TG) levels increased in the mixed beer group by 16.89 mg/dL (*p* = 0.32) and in the wheat beer group by 15.27 mg/dL (*p* = 0.32). They decreased in the water and pilsener groups, although these changes were not statistically significant (Figure 1E, Supplementary Table S1). The relative difference (TG value at week 4/TG value at week 0) was significantly higher in subjects drinking wheat beer than in subjects drinking water (*p* = 0.036, Wilcoxon signed rank test on paired samples). The cholesterol and LDL cholesterol increased in the mixed beer group by 8.22 mg/dL (*p* = 0.2) and 7.37 mg/dL (*p* = 0.12), respectively, and decreased in the pilsener and water groups (Figure 1F,G, Supplementary Table S1). No changes could be observed in the wheat beer group (Figure 1E–G, Supplementary Table S1). Simultaneously, HDL cholesterol levels increased in the water group and decreased in the wheat beer group, but were not affected by mixed beer or pilsener consumption (Figure 1H, Supplementary Table S1).

Adiponectin is an important cytokine derived from adipose tissue that has the capability to reduce hepatic and systemic insulin resistance and reduce inflammation and fibrosis in the liver. Adiponectin levels were higher in the water, pilsener, and mixed beer groups, though these variations were not statistically significant; no changes were observed in the wheat beer group (Supplementary Figure S1A).

The results indicate that wheat beer and mixed beer modulate the glucose and fat metabolism, while water and pilsener seem to have a beneficial effect on the fat metabolism.

### 3.2. Impact of Water, Non-Alcoholic Pilsener, and Non-Alcoholic Mixed Beer on Liver Damage

**Figure 2:**
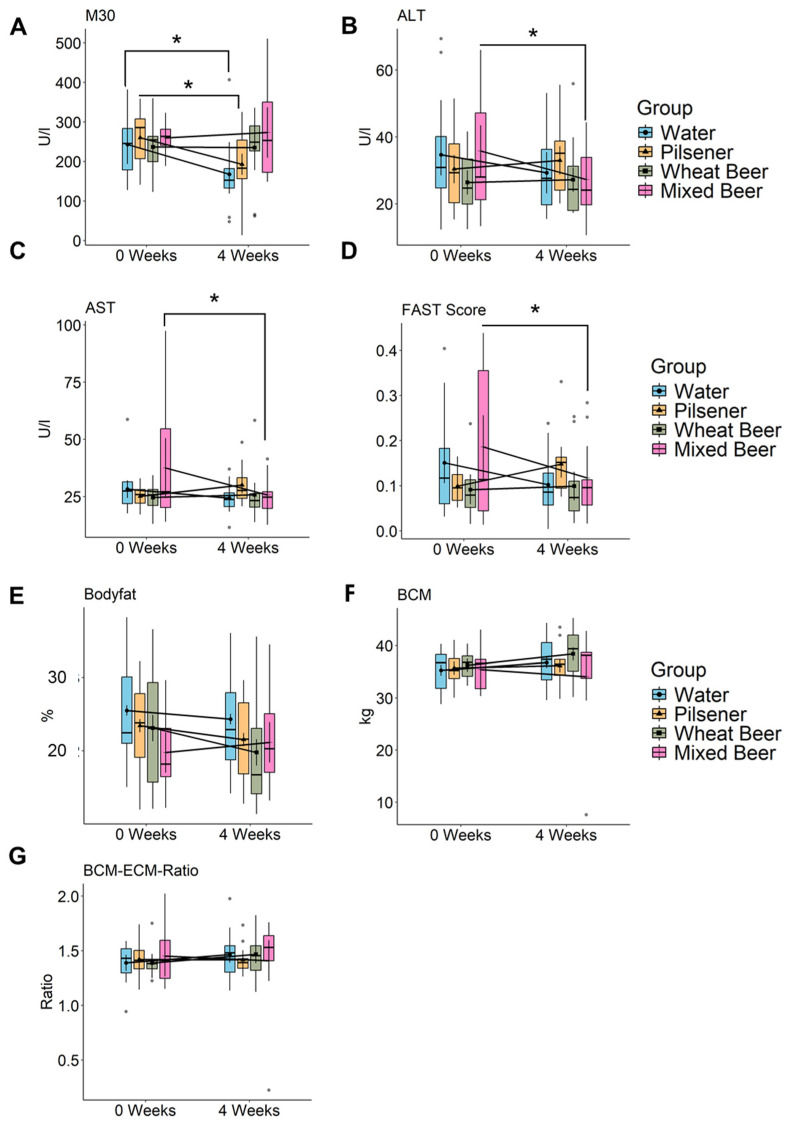
Non-alcoholic beer consumption alters liver damage markers and changes body composition. Markers of liver damage were measured, and bioelectrical impedance analysis (BIA) was performed in healthy young men at baseline and after 4 weeks. Estimated marginal means and error bars (95% confidence interval) are plotted in the foreground and raw data are plotted in the background. Significance was calculated using two-way repeated measures ANOVA. Significant difference between time points using pairwise comparison of estimated marginal means is marked in the plot (* *p* < 0.05). (**A**) M30 (WA: *p* = 0.0162, PI: *p* = 0.0373, WB: *p* = 0.9563, MB: *p* = 0.69), (**B**) ALT (WA: *p* = 0.11, PI: *p* = 0.46, WB: *p* = 0.82, MB: *p* = 0.03), (**C**) AST (WA: *p* = 0.31, PI: *p* = 0.24, WB: *p* = 0.78, MB: *p* = 0.013), (**D**) FAST (WA: *p* = 0.075, PI: *p* = 0.086, WB: *p* = 0.77, MB: *p* = 0.032), (**E**) Body fat (WA: *p* = 0.24, PI: *p* = 0.076, WB: *p* = 0.0026, MB: *p* = 0.21), (**F**) BCM (WA: *p* = 0.58, PI: *p* = 0.52, WB: *p* = 0.1, MB: *p* = 0.23), and (**G**) BCM ECM Ratio. ALT = alanine transaminase, AST = Aspartate transaminase, FAST = Fibroscan-AST Score, and BCM = body cell mass.

The apoptosis marker M30, a surrogate marker for the apoptosis of epithelial cells (i.e., hepatocytes), decreased statistically significantly in the water and pilsener groups and increased slightly in the mixed beer group; no changes were detected in the wheat beer group (Figure 2A).

In contrast, liver enzymes ALT and AST statistically significantly decreased in the mixed beer and water groups. In the pilsener group, ALT and AST showed a slight increase (Figure 2B,C). In line with this, the FAST Score, combining the liver stiffness measurement (LSM, control attenuated parameter (CAP)) and AST, decreased in the water group, and statistically significantly in the mixed beer group, but increased in the pilsener group (Figure 2D).

The relative difference in AST (AST at week 0 to AST at week 4) and the FAST Score was statistically significantly higher in the pilsener group compared to the water group. No changes were observed in the wheat beer group regarding M30, ALT, AST, or the FAST Score (Figure 2).

In summary, pilsener and water consumption led to a reduction in M30, alongside an increase in transaminases and the FAST Score. Conversely, the mixed beer group had high M30 and low ALT and AST and FAST Score.

### 3.3. Liver Steatosis and Stiffness After Abstinence from Drinks Other than Water After 4 Weeks

Steatosis and liver stiffness were assessed using transient elastography. The CAP score for steatosis decreased in the water and wheat beer groups, while no differences were observed for the mixed beer and pilsener groups (Supplementary Figure S1B,C). Liver stiffness decreased in the water group but increased in all other groups, with values remaining in the normal range (Supplementary Figure S1B,C).

### 3.4. Wheat and Mixed Beer Influence Body Composition

We performed BIA measurement to determine body composition. After wheat beer consumption, the body fat percentage statistically significantly decreased (Figure 2E). In the pilsener and water groups, the body fat also showed a decreasing but statistically non-significant trend, while the trend was increasing in the mixed beer group, which was also not statistically significant (Figure 2E, Supplementary Table S1). The body cell mass (BCM), representing metabolically active tissues including muscle cells, organs, blood, and immune cells, increased in the water, pilsener, and wheat beer groups (Figure 2F). The extracellular mass (ECM) to BCM ratio, which indicates malnutrition when elevated, increased in the mixed beer group, but did not affect any other group (Figure 2G, Supplementary Table S1).

### 3.5. Pilsener Consumption Decreased Alpha Diversity and Firmicutes Abundance

**Figure 3:**
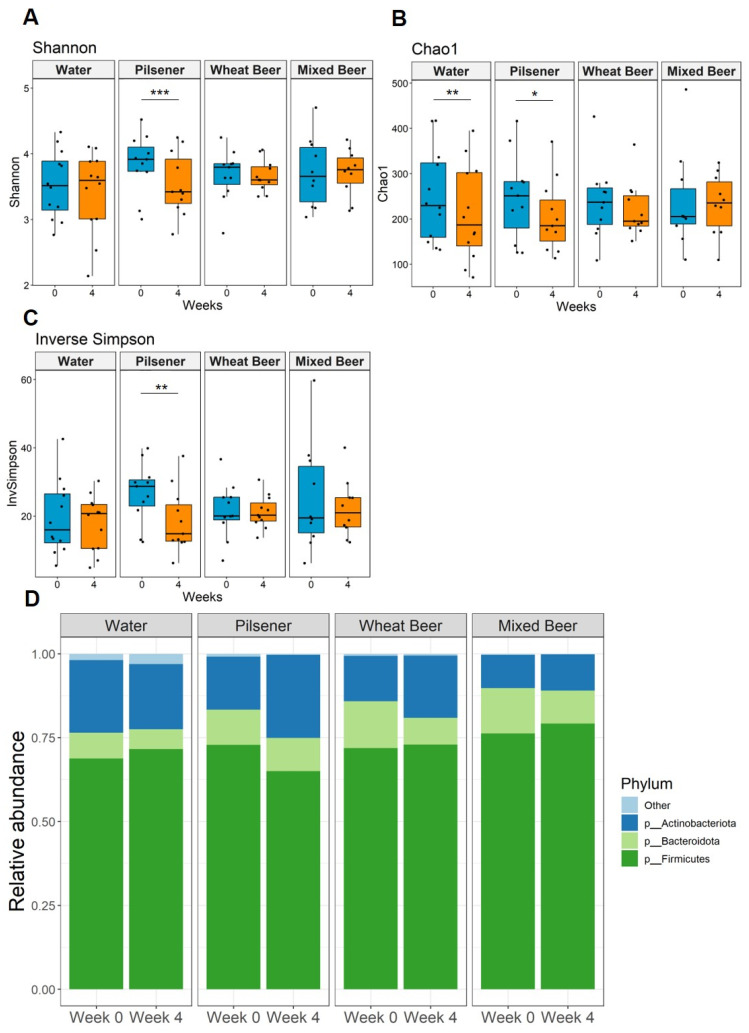
Non-alcoholic beer consumption changes alpha diversity and alters microbiota composition at the phyla level. Fecal samples were collected at baseline and after 4 weeks. Significance was calculated with Wilcoxon signed rank test on paired samples (* *p* < 0.05; ** *p* < 0.01; *** *P* < 0.001) (**A**–**C**) We detected 7237 species among all groups. Microbial diversity based on the (**A**) Shannon, (**B**) Chao 1, and (**C**) inverse Simpson indices. (**D**) Relative abundance of bacteria per group and time point summarized at the phylum level.

We detected a total of 7237 different species among the whole cohort. Bacterial diversity statistically significantly decreased in patients consuming pilsener (Figure 3A–C). Diversity also statistically significantly decreased in the water group, as indicated by Chao1 (Figure 3B). No statistically significant changes were observed in subjects drinking wheat beer or mixed beer (Figure 3A–C). Beta diversity analysis revealed no statistically significant difference between week 0 and week 4 (Supplementary Figure S2A–D).

**Figure 4:**
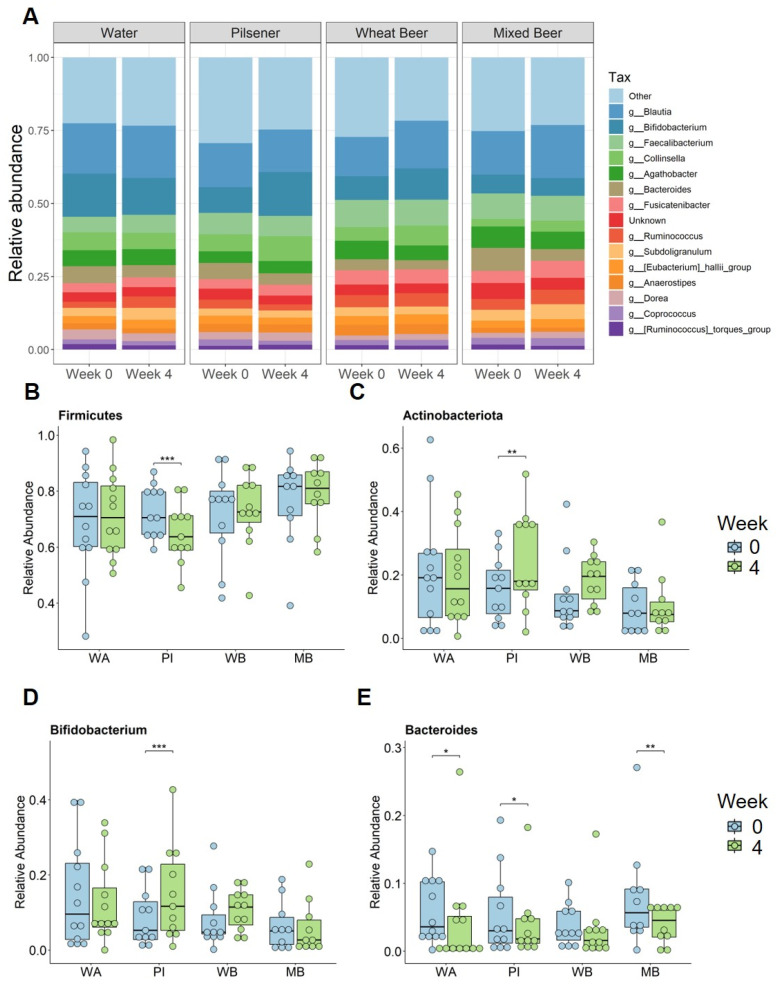
Non-alcoholic beer consumption changes microbiota composition on the genera level. Fecal samples were collected at baseline and after 4 weeks. Significance was calculated with Wilcoxon signed rank test on paired samples (* *p* < 0.05; ** *p* < 0.01; *** *p* < 0.001). (**A**) Relative abundance of bacteria per group and time point summarized at the genera level. Relative abundance for (**B**) *Firmicutes*, (**C**) *Actinobacteriota*, (**D**) *Bifidobacterium*, and (**E**) *Bacteroides* at baseline and after 4 weeks for each group. WA = water, PI = pilsener, WB = wheat beer, and MB = mixed beer.

At the phylum level, *Firmicutes* were the main phylum across all groups and time points, followed by *Bacteroidota* and *Actinobacteriota* (Figure 3D). In the pilsener group, the relative abundance of *Firmicutes* statistically significantly decreased, while the abundance of *Actinobacteriota* increased statistically significantly (Figure 3D and Figure 4B,C). In contrast, the relative abundance of *Bacteroidota* statistically significantly decreased in the water and wheat beer groups. The relative abundance of *Actinobacteriota* also increased in the wheat beer group (Figure 3D and Figure 4B).

At the genus level, we focused on bacteria with a prevalence greater than 70%. The *Actinobacteriota* genera *Bifidobacterium* and *Collinsella* statistically significantly increased in the pilsener group (Figure 4A,D). *Collnisella* also statistically significantly increased in the mixed beer and wheat beer groups (Figure 4A). The most prevalent genus of *Bacteroidota Bacteroides* statistically significantly decreased in all groups (except the wheat beer group) (Figure 4E).

Among the *Firmicutes* genera, *Ruminococcus* and *Subdoligranulum* increased statistically significantly in the water and mixed beer groups, while *Ruminococcus* decreased in the pilsener group (Figure 4A). *Dorea* increased statistically significantly in the wheat beer and mixed beer groups, whereas it decreased in the water group (Figure 4A). *Blautia* increased statistically significantly in the wheat beer and mixed beer groups (Figure 4A).

**Figure 5:**
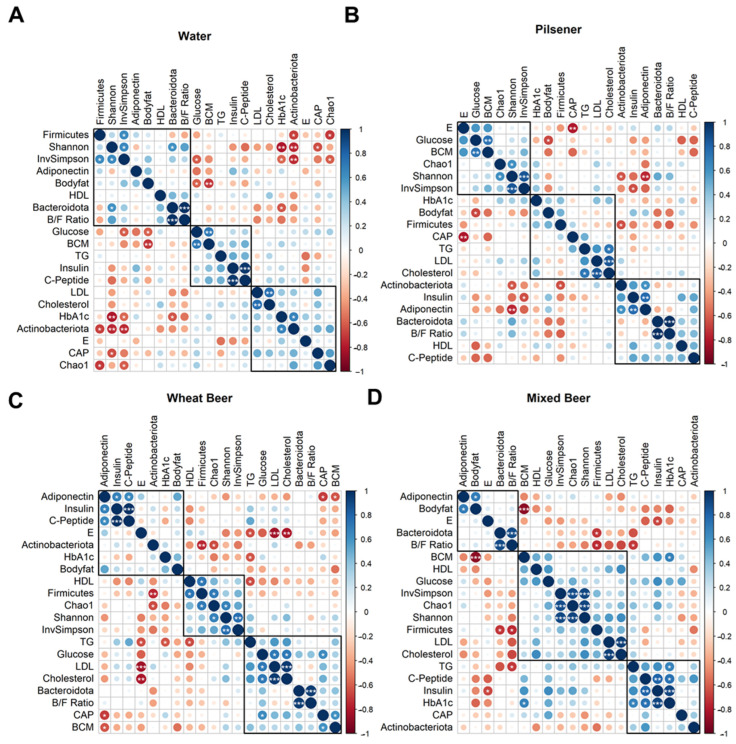
Changes in the parameters are clustered differently among groups. For each included parameter, the difference between week 4 and baseline was calculated and correlated with each other separately for each group ((**A**): Water, (**B**): pilsener, (**C**): wheat beer, (**D**): mixed beer) using Pearson’s correlation and hierarchical clustering (* *p* < 0.05; ** *p* < 0.01; *** *p* < 0.001). Clusters indicate similarity of changes in the parameter per group. B/F ratio: Bacteroides–Firmicutes ratio, BCM: body cell mass, CAP: controlled attenuation parameter, E: liver stiffness, HDL: high-density lipoprotein, LDL: low-density lipoprotein, and TG: triglycerides.

Finally, correlations between the changes in each parameter were analyzed across the groups (Figure 5). In the mixed beer group, changes in adiponectin and body fat were positively correlated (*R* = 0.72, *p* = 0.027) (Figure 5D). In all other groups, adiponectin was positively correlated with insulin and C-peptide (Figure 5A–C, Supplementary Tables S2–S5).

## 4. Discussion

This study investigates the effects of NAB on glucose and fat metabolism, body composition, and microbiota composition in healthy young men. Our findings indicate that the impact of these beverages varies depending on their ingredients. Specifically, non-alcoholic pilsener altered the microbiota composition by increasing *Actinobacteriota* at the expense of *Firmicutes*, but did not affect fat metabolism or body composition.

M30 is cleaved from the type-I intermediate filament cytokeratin 18 and released into the blood upon apoptosis. It is expressed in all epithelial cells, but highly enriched in hepatocytes, where it accounts for 5% of all protein content. Elevated M30 levels have been linked to DILI, liver inflammation, and steatosis. Its accuracy for apoptosis in hepatocytes is higher than in ALT [18,19,20].

Previous research has shown that lager-style NAB consumption leads to reductions in fasting blood glucose and an increase in functional beta cells after 30 days, with corresponding decreases in ALT and AST levels [9]. By contrast, our study did not find similar effects in the pilsener-style group, but we did observe a decrease in the liver apoptosis biomarker M30, indicating decreased liver damage. Furthermore, consumption of mixed beer decreased AST, ALT, and the FAST Score. When comparing the raw data for ALT, AST, and the FAST Score, we observed three outliers, which exert a disproportionate influence on the mean, thereby limiting its reliability as a measure of central tendency in this context.

These variations may be attributed to variations in study design, subject demographics (including diet, sex, and age), and regional differences. Additionally, neither our study nor the previous study controlled for alcohol use by the subjects prior to entering the study. As such, the reductions in liver enzymes yet increase in M30 levels seen among subjects in the mixed beer group could be explained in part, by alcohol abstinence prior to the study.

Although alcohol has been removed from the NAB variants, these drinks still contain a considerable number of calories and sugar, which is not transformed into ethanol (Table 2). High-calorie drinks contribute to weight gain and promote the progression of metabolism-associated diseases such as obesity, diabetes, and metabolic syndrome. Sweet drinks are especially involved in the development and progression of metabolic dysfunction-associated steatotic liver disease (MASLD) [21,22]. Our study found that the daily consumption of non-alcoholic mixed beer increased fasting glucose, and non-alcoholic wheat beer increased insulin levels, thus indicating the impairment of glucose metabolism and development of insulin resistance—a key component of metabolic syndrome and MASLD. Additionally, subjects drinking pilsener beer showed a significant increase in HbA1c, while mixed beer and wheat beer only showed a slight increase. However, HbA1c reflects the average blood glucose levels over the preceding three months and is therefore not exclusively influenced by changes occurring within the last four weeks.

High-sugar-containing drinks have been linked to disruption in fat metabolism [23], and our data demonstrate that non-alcoholic mixed beer consumption shows an increasing trend for TG, cholesterol, and LDL cholesterol levels. Elevated cholesterol and LDL cholesterol are markers of metabolic syndrome and are associated with increased risk of cardiovascular diseases. In contrast, non-alcoholic pilsener intake reduced total cholesterol and LDL cholesterol levels, but did not show significance. In the water group, a decrease in LDL cholesterol was observed, which might be due to the abstinence from sugar and alcohol-containing drinks. Additionally, no consumption of NAB (the group consuming water) increased HDL cholesterol, which is protective against cardiovascular disease, and decreased HDL levels were described with increased liver damage in patients with acute liver injury [24]. Wheat beer did not affect fat metabolism.

Polyphenols are secondary plant metabolites found in both alcoholic and non-alcoholic beer. They are more concentrated in wheat beer than pilsner or non-alcoholic beer. Beer is particularly enriched with polyphenols such as Naringenin, Catechin, Quercetin, Rutin, Arbutin, and Berberine, which show various biological effects, including antioxidant and anti-inflammatory properties [25,26,27]. In this study, the non-alcoholic mixed beer group showed an increase in TG, cholesterol, and LDL cholesterol, indicating that the effects of sugar and a high calorie intake outweigh the potential impact of polyphenols on lipid metabolism. Additionally, fasting glucose, insulin, and C-peptide increased in the non-alcoholic mixed beer and non-alcoholic wheat beer groups, but not in the non-alcoholic pilsener and water groups. Cholesterol and LDL cholesterol also decreased in the water and pilsener groups. Given that the effects of water and pilsener were identical, and water contains no polyphenols, we infer that polyphenols did not compensate for the adverse effects of NAB on lipid and glucose metabolism in this study.

These findings align with previous studies on the consumption of NA beverages, which reported no notable changes in cholesterol or TG levels in human or mice independent of their BMI [9,13,28,29].

However, these studies reported effects in people with moderate alcohol consumption. The authors linked the effect to polyphenols found in both AB and NAB. Another study administered commercial hop at 400 mg/d to 29 women aged 58–73 after a 6-month episode of abstinence. After 30 days, significant reductions in total cholesterol, oxidized LDL, and TG were observed [13]. In the same group of women, the administration of NAB did not show the same effect, indicating that the dose, the wash-out phase, or other components of NAB counteracting this effect are important criteria [13]. Furthermore, polyphenols in non-alcoholic products are less well absorbed than in alcoholic-products [30]. Even though the protective effect of polyphenols in beer on cardiovascular events has been shown, this effects the anti-inflammatory effect. The improvement in the lipid profile has been linked to the alcohol fraction [31,32].

In conclusion, our results indicate that the observed effects on lipid and glucose metabolism are primarily driven by calorie intake, rather than the effect of polyphenols, as they are not that abundant in NAB and not well absorbed (see Table 2 for polyphenols amounts in NAB).

Adiponectin is an adipokine from adipose tissue that has an anti-diabetic, insulin-sensitizing, anti-atherogenic, and anti-inflammatory function. High adiponectin levels are associated with reduced risk of myocardial infarction, but paradoxically, they have also been linked to an increased risk of stroke and higher cardiovascular and all-cause mortality [33,34]. Adiponectin levels in obese patients also correlate with adipocyte size in visceral adipose tissue, and with increased adipocyte size, the risk of liver damage increases [35,36]. This paradox is thought to be due to adiponectin resistance or a compensatory response. In our study, adiponectin levels increased in the water, pilsener, and mixed beer groups. We expected a decrease in adiponectin in the mixed beer group due to the high sugar content, but observed an increase, which was positively associated with increased body fat, but may also hint towards an increased risk of liver damage. It is likely that other unmeasured factors influenced the adiponectin levels.

Polyphenols also affect gut microbiota. Previous studies have shown that NAB consumption for 30 days increased *Bacteroides* and decreased *Firmicutes* [9]. In our study, pilsener consumption led to a decrease in *Firmicutes* and an increase in *Actinobacteria*, while wheat beer and water consumption increased *Actinobacteria* at the expense of *Bacteroidetes.* Alpha diversity in the pilsener group significantly decreased, which contrasts with findings in other studies, where NAB consumption either increased alpha diversity or showed no effect [9,11].

Additionally, digested pilsener beer has been shown to expand the *Bifidobacterium* population, which is beneficial to human health [37]. Ferulic acid amplifies alpha diversity and increases short-chain fatty acid production, while Quercetin has been shown to improve dysbiosis by elevating the *Firmicutes* to *Bacteroidetes* ratio and decreasing obesity-associated microbiota species in rats [38,39]. Our study observed a significant increase in the *Bifidobacterium* fraction with pilsener, but no effect on the *Firmicutes/Bacteroides* ratio. The consumption of mixed beer and wheat beer reduced this ratio. Interestingly, two participants with an initial *Firmicutes/Bacteroides* ratio greater than one showed improvements, with their post-intervention ratio dropping below one. While polyphenols may alter the gut microbiota, other factors also contribute to these changes, and the microbiome in healthy individuals typically remains stable, returning to its baseline after short-term disturbances. Even though we included a wash-out period before the intervention, previous alcohol consumption might affect the changes in the intestinal microbiome.

This study has limitations, including the lack of dietary control and its focus on healthy young men, which limits the generalizability of the results to other demographics, such as women, older individuals, or those with underlying diseases. Another limitation is that this study was conducted with a rather small cohort. Even though we performed a power analysis in advance to determine an ideal group size, we did not reach the desired number of subjects. This was due to the limitations and restrictions of the study due to the global COVID-19 pandemic. Due to slow recruitment, we extended the strict inclusion criteria of a limited BMI from a maximum of 25 kg/m^2^ to a maximum of 30 kg/m^2^ to increase the size of the study cohort. We were of course aware that dyslipidemia or elevated blood lipid levels can also occur from a BMI > 25 kg/m^2^, but we did not see a correlation of increased BMI with increased blood lipids or even dyslipidemia in our study cohort. Due to the small number of groups, many results only showed trends and did not reach statistical significance. It would also have been interesting if we could have included regular alcoholic beer as a kind of positive control group in the overall context, but this group could not be initiated for ethical reasons, as our local ethics committee had concerns about inducing addictive behavior in the subjects through study-associated daily alcohol consumption. The comparison with alcoholic beer would have given our results more power, but the ethics committee’s objection was completely justified. For future studies, more attention should be paid to these aspects for more reliable and reproducible study results in real-life cohorts. The baseline characteristics show differences in the mixed and wheat beer groups that might affect the outcomes. Additionally, the long-term consequences of NAB consumption remain unclear, as our study was conducted over a short 4-week period.

## 5. Conclusions

In summary, the consumption of non-alcoholic beverages has unfavorable effects on metabolism, mainly driven by their calorie and sugar contents. The small residual alcohol content (up to 0.5%) may also contribute to these outcomes. Non-alcoholic pilsener has fewer adverse effects compared to other drinks, but no benefit when compared to no consumption of NAB.

## Figures and Tables

**Table 1:** Clinical characteristics of subjects consuming water, pilsener, wheat beer, or mixed beer. Values are presented as median and interquartile range in parentheses. The number of patients for whom data were available is indicated in the first column. *p*-values were calculated with Kruskal–Wallis rank sum test for continuous variables and Exact Fisher’s Test for categorical variables. BMI, body mass index; AP, Alkaline phosphatase; ALT, alanine aminotransferase; AST, aspartate aminotransferase; CAP, controlled attenuation parameter (liver steatosis); GGT, gamma-glutamyl-transferase; HbA1c, glycated hemoglobin A1c; HDL, high-density lipoprotein; LDH, lactate dehydrogenase; LDL, low-density lipoprotein; and LSM, liver stiffness measurement.

Characteristic	Overall n = 44	Water n = 12	Pilsener n = 11	Wheat Beer n = 11	Mixed Beer n = 10	*p*-Value
**Body Characteristics**						
Height (cm) n = 44	183.0 (177.5, 186.5)	183.0 (177.0, 185.5)	183.0 (174.0, 190.0)	185.0 (180.0, 186.0)	179.0 (178.0, 187.0)	0.8
Weight (kg) n = 44	80 (73, 88)	81 (77, 89)	79 (72, 90)	80 (74, 94)	74 (68, 83)	0.4
BMI (kg/m^2^) n = 44	24.5 (22.1, 26.0)	24.8 (24.1, 26.2)	24.8 (21.6, 27.1)	23.8 (21.4, 29.1)	22.8 (21.4, 25.9)	0.5
Waist Circumference (cm) n = 44	83 (78, 88)	85 (80, 86)	86 (76, 96)	85 (75, 99)	79 (76, 83)	0.3
Hip Circumference (cm) n = 44	94.5 (91.5, 98.0)	97.0 (93.0, 100.5)	95.0 (91.0, 97.0)	94.0 (90.0, 98.0)	92.0 (91.0, 96.0)	0.4
Body fat (kg) n = 44	18 (13, 24)	19 (17, 26)	18 (13, 26)	18 (11, 32)	14 (11, 20)	0.3
Body Cell Mass (BCM) (kg) n = 44	36.3 (32.8, 38.0)	36.7 (31.4, 38.5)	35.2 (33.3, 37.7)	36.8 (33.2, 38.2)	36.6 (31.8, 37.5)	>0.9
Extracellular Mass (ECM) (kg) n = 44	25.7 (24.0, 27.6)	26.1 (23.6, 27.1)	26.0 (22.4, 28.2)	26.5 (25.6, 28.5)	24.9 (23.9, 25.7)	0.5
LSM (kPa) n = 44	5.05 (3.2, 7.1)	5.3 (3.7, 7.1)	5.05 (3.5, 5.2)	4.49 (3.3, 6.3)	5.34 (3.2, 5.2)	0.3
Liver steatosis CAP (db/m^2^) n = 44	218.8 (152, 354)	226.6 (181, 354)	216.9 (162, 291)	218.2 (152, 287)	213.2 (172, 318)	>0.9
**Laboratory parameters**						
Bilirubin (mg/dL) n = 43	0.76 (0.60, 0.95)	0.88 (0.75, 1.24)	0.70 (0.47, 1.10)	0.74 (0.60, 0.78)	0.73 (0.57, 0.90)	0.2
Creatinine (mg/dL) n = 43	0.88 (0.82, 0.98)	0.94 (0.86, 1.00)	0.86 (0.77, 0.91)	0.91 (0.85, 0.99)	0.85 (0.79, 0.92)	0.2
AST (U/L) n = 43	27 (21, 30)	27 (21, 32)	26 (22, 29)	26 (21, 29)	27 (20, 55)	0.9
ALT (U/L) n = 43	28 (19, 42)	31 (23, 44)	29 (19, 44)	25 (16, 34)	28 (21, 47)	0.8
AP (U/L) n = 43	74 (64, 90)	78 (63, 93)	77 (73, 94)	65 (59, 88)	69 (67, 87)	0.5
GGT (U/L) n = 43	17 (14, 23)	20 (15, 23)	20 (14, 28)	15 (12, 17)	18 (16, 20)	0.4
LDH (U/L) n = 43	166 (157, 177)	161 (154, 180)	172 (166, 175)	162 (154, 172)	171 (164, 191)	0.3
Lipase (U/L) n = 43	28 (23, 39)	28 (21, 42)	27 (23, 38)	27 (22, 31)	30 (28, 39)	0.8
Albumin (g/dL) n = 43	48.10 (44.90, 49.60)	46.90 (44.10, 49.65)	48.80 (44.30, 50.50)	48.10 (44.50, 49.20)	48.10 (45.80, 49.20)	0.8
M30 (U/L) n = 43	255 (198, 286)	245 (177, 286)	286 (198, 316)	254 (197, 268)	264 (241, 281)	0.6
Adiponectin (µg/L) n = 43	5.1 (4.2, 9.1)	4.8 (3.6, 8.4)	5.1 (3.5, 7.5)	7.5 (4.8, 10.1)	5.1 (4.8, 6.5)	0.5
**Glucose metabolism**						
Fasting glucose (mg/dL) n = 43	84 (81, 90)	86 (82, 91)	85 (76, 94)	83 (78, 88)	84 (81, 84)	0.9
C-Peptide (nmol/L) n = 43	0.56 (0.45, 0.67)	0.48 (0.44, 0.69)	0.55 (0.45, 0.65)	0.50 (0.43, 0.70)	0.59 (0.57, 0.62)	0.9
HbA1c (%) n = 43	5.00 (4.80, 5.20)	5.00 (4.90, 5.10)	4.90 (4.70, 5.10)	5.10 (4.90, 5.20)	4.90 (4.90, 5.10)	0.5
Insulin (µU/mL) n = 43	7.4 (5.4, 10.2)	7.4 (5.7, 9.5)	8.0 (5.6, 9.1)	7.4 (4.3, 11.9)	7.0 (6.6, 9.3)	>0.9
**Fat metabolism**						
Cholesterol (mg/dL) n = 43	167 (148, 183)	166 (149, 191)	168 (145, 200)	160 (148, 183)	169 (160, 183)	>0.9
Triglycerides (mg/dL) n = 43	69 (56, 106)	83 (66, 116)	72 (56, 98)	59 (48, 76)	65 (56, 69)	0.3
HDL-Cholesterol (mg/dL) n = 43	53 (47, 61)	53 (42, 59)	52 (45, 55)	55 (49, 64)	51 (49, 60)	0.6
LDL-Cholesterol (mg/dL) n = 43	91 (77, 113)	99 (77, 116)	84 (65, 129)	88 (69, 98)	95 (81, 116)	0.7

**Table 2:** Calorie, sugar, alcohol, and polyphenol content of the included non-alcoholic beverages; n/a = not available.

	Wheat Beer		Pilsener		Mixed Beer	
	100 mL	660 mL	100 mL	660 mL	100 mL	660 mL
Calories (kJ)	107.0	706.2	111.0	732.6	114.0	752.4
Fat (g)	0.1	0.66	0.0	0.0	0.0	0.0
Carbohydrates (g)	5.3	34.98	5.7	37.62	6.3	41.58
Sugar (g)	3.6	23.76	2.8	18.48	4.9	32.34
Protein (g)	0.4	2.64	0.0	0.0	0.0	0.0
Alcohol (%)	<0.5	<0.5	<0.5	<0.5	<0.5	<0.5
Polyphenols (mg)	30	198	n/a	n/a	n/a	n/a

## Data Availability

The data presented in this study are available on request from the corresponding author. The data are not publicly available due to privacy and ethical reasons.

## Supplementary Materials

The following supporting information can be downloaded at: https://www.mdpi.com/article/10.3390/nu17101625/s1. Supplementary Table S1: Results of repeated measurements ANOVA from Figures 1A and 2G. Supplementary Table S2: Correlation Matrix Water. Supplementary Table S3: Correlation Matrix pilsener. Supplementary Table S4: Correlation Matrix Wheat Beer. Supplementary Table S5: Correlation Matrix Mixed Beer. Supplementary Table S6: Alpha diversity as calculated by Shannon, Chao1 and Inverse Simpson Index at week 0 and week 4 for all groups. Supplementary Table S7: Relative Abundance for selected bacteria at week 0 and week 4 for all groups. Supplementary Figure S1: Non-alcoholic beer consumption did not alter liver stiffness or liver steatosis as measured by transient elastography. Supplementary Figure S2: Beta diversity did not change after 4 weeks consumption of non-alcoholic beers. Supplementary Figure S3: Dendrogram for hierarchical clustering in correlation matrix from Figure 5.

## Abbreviations

Alanine transferase, ALT; Alcohol Use Disorders Identification Test questionnaire, AUDIT; aspartate transferase, AST; alcoholic beer, AB; bioelectrical impedance analysis, BIA; body cell mass, BCM; controlled attenuated parameter, CAP; extracellular mass, ECM; Fibroscan-AST, FAST; hemoglobin A1c, HbA1c; high-density lipoprotein, HDL; liver stiffness measurement, LSM; low-density lipoprotein, LDL; NAB, non-alcoholic beer; metabolic dysfunction-associated steatotic liver disease, MASLD; transient elastography measurement, TEM; triglycerides, TGs; and WHO, World Health Organization.
