# Supplementary material for: Non-Alcoholic Beer Influences Glucose and Lipid Metabolism and Changes Body Composition in Healthy, Young, Male Adults
Source: Nutrients. 2025 May 9;17(10):1625. doi: 10.3390/nu17101625 (PMC12114185; doi:10.3390/nu17101625)
Supplement: Supplementary file 1 [file nutrients-17-01625-s001.zip › nutrients-3563941-supplementary.pdf]

Supplementary Data

Supplementary Tables

Supplementary Table S1: Results of repeated measurements ANOVA from Figure 1A-2G.

	df	*MSE*	*F*	*ges*	*p-value*
Insulin					
Group	3. 39	27.21	0.62	0.030	0.604
Time	1. 39	14.39	1.95	0.017	0.171
Interaction	3. 39	14.39	2.02	0.051	0.127
C-Peptide					
Group	3. 39	0.06	0.35	0.021	0.788
Time	1. 39	0.01	5.76	0.028	*0.021*
Interaction	3. 39	0.01	1.23	0.018	0.310
Glucose					
Group	3. 39	110.53	0.18	0.011	0.931
Time	1. 39	25.49	5	0.023	*0.031*
Interaction	3. 39	25.49	1.04	0.015	0.384
HbA1c					
Group	3. 39	0.15	0.1	0.007	0.959
Time	1. 39	0.01	10.96	0.022	*0.002*
Interaction	3. 39	0.01	1.92	0.012	0.142
Triglycerides					
Group	3. 38	4264.97	0.06	0.004	0.98
Time	1. 38	730.75	0.91	0.003	0.346
Interaction	3. 38	730.75	1.16	0.013	0.339
Cholesterol					
Group	3. 39	2713.76	0.27	0.19	0.845
Time	1. 39	182.64	0.02	<0.001	0.877
Interaction	3. 39	182.64	1.07	0.005	0.371
LDL					
Group	3. 39	2184.01	0.34	0.24	0.798
Time	1. 39	98.66	0.07	<0.001	0.8
Interaction	3. 39	98.66	1.03	0.003	0.39
HDL					
Group	3. 39	389.71	0.17	0.012	0.917
Time	1. 39	21.29	0.29	<0.001	0.562
Interaction	3. 39	21.29	1.56	0.006	0.214
M30					
Group	3. 39	8566.88	1.53	0.067	0.222
Time	1. 39	5355.35	4.22	0.04	*0.047*
Interaction	3. 39	5355.35	2	0.056	0.13
ALT					
Group	3. 39	305.75	0.44	0.027	0.726
Time	1. 39	64.66	2.27	0.01	0.140

Interaction	3. 39	64.66	2.15	0.028	0.110
AST					
Group	3. 39	190.14	0.8	0.04	0.502
Time	1. 39	91.82	1.39	0.011	0.245
Interaction	3. 39	91.82	2.8	0.065	0.053
FAST Score					
Group	3. 39	0.01	0.81	0.045	0.494
Time	1. 39	0.00	1.13	0.007	0.294
Interaction	3. 39	0.00	3.54	0.062	*0.023*
Bodyfat					
Group	3. 40	0.01	0.88	0.059	0.457
Time	1. 40	0.00	5.82	0.009	*0.021*
Interaction	3. 40	0.00	3.44	0.015	*0.026*
BCM					
Group	3. 40	41.85	0.57	0.034	0.639
Time	1. 40	9.58	1.04	0.005	0.314
Interaction	3. 40	9.58	1.29	0.018	0.290
BCM/ECM Ratio					
Group	3. 40	0.09	0.01	<0.001	0.999
Time	1. 40	0.03	0.61	0.004	0.441
Interaction	3. 40	0.03	0.72	0.013	0.544

Supplementary Table S2: Correlation Matrix Water. Data for Figure 5A. Correlation of delta for different variables for subjects consuming water. First Table shows the correlation coefficient and the second table the p-value. Correlation was calculated with Pearson. B/F Ratio: Bacteroides-Firmicutes-Ratio, BCM: Body cell mass, CAP: Controlled attenuation parameter, E: Liver stiffness, HDL: high density lipoprotein, LDL: low density lipoprotein

R^2	Glucose	Insulin	C-Peptide	HbA1c	Cholesterol	Triglyceride	HDL	LDL	Bodyfat	BCM	Adiponectin	E	CAP	Shannon	Chao1	InvSimpson	Firmicutes	Bacteroidota	B/F Ratio	Actinobacteriota
Glucose	1.00	0.27	0.31	0.17	0.01	0.01	0.22	-0.19	-0.59	0.72	-0.53	0.25	0.33	-0.07	0.35	-0.60	-0.39	0.05	0.42	0.20
Insulin	0.27	1.00	0.96	0.26	0.21	0.51	0.19	-0.20	-0.10	0.41	0.45	-0.48	0.19	-0.44	0.11	-0.24	0.06	-0.21	0.02	0.11
C-Peptide	0.31	0.96	1.00	0.32	0.31	0.47	0.25	-0.11	-0.17	0.49	0.34	-0.39	0.33	-0.56	0.19	-0.35	-0.09	-0.18	0.01	0.22
HbA1c	0.17	0.26	0.32	1.00	0.31	-0.05	-0.26	0.38	0.23	0.32	0.24	0.36	0.33	-0.81	0.12	-0.58	-0.19	-0.62	-0.35	0.67
Cholesterol	0.01	0.21	0.31	0.31	1.00	0.30	0.37	0.76	0.35	0.19	-0.21	-0.15	0.43	-0.43	0.13	-0.10	0.02	-0.31	0.05	0.23
Triglyceride	0.01	0.51	0.47	-0.05	0.30	1.00	-0.04	-0.21	-0.07	0.32	0.08	-0.55	-0.38	-0.02	-0.15	0.05	0.15	-0.17	0.04	-0.05
HDL	0.22	0.19	0.25	-0.26	0.37	-0.04	1.00	-0.03	0.09	-0.07	-0.09	-0.13	0.27	0.10	0.24	0.06	0.06	0.46	0.50	-0.45
LDL	-0.19	-0.20	-0.11	0.38	0.76	-0.21	-0.03	1.00	0.38	0.01	-0.25	0.10	0.50	-0.41	-0.03	-0.04	0.03	-0.45	-0.23	0.38
Bodyfat	-0.59	-0.10	-0.17	0.23	0.35	-0.07	0.09	0.38	1.00	-0.62	0.50	-0.04	-0.16	-0.11	0.02	0.31	0.11	-0.03	0.10	0.04
BCM	0.72	0.41	0.49	0.32	0.19	0.32	-0.07	0.01	-0.62	1.00	-0.40	0.07	0.15	-0.19	-0.10	-0.47	-0.15	-0.27	0.00	0.22
Adiponectin	-0.53	0.45	0.34	0.24	-0.21	0.08	-0.09	-0.25	0.50	-0.40	1.00	-0.33	-0.21	-0.28	-0.10	0.15	0.27	-0.16	-0.30	-0.05
E	0.25	-0.48	-0.39	0.36	-0.15	-0.55	-0.13	0.10	-0.04	0.07	-0.33	1.00	0.14	-0.03	0.13	-0.12	-0.28	0.15	-0.02	0.18
CAP	0.33	0.19	0.33	0.33	0.43	-0.38	0.27	0.50	-0.16	0.15	-0.21	0.14	1.00	-0.65	0.56	-0.52	-0.26	-0.28	-0.15	0.47
Shannon	-0.07	-0.44	-0.56	-0.81	-0.43	-0.02	0.10	-0.41	-0.11	-0.19	-0.28	-0.03	-0.65	1.00	-0.37	0.65	0.28	0.60	0.40	-0.75
Chao1	0.35	0.11	0.19	0.12	0.13	-0.15	0.24	-0.03	0.02	-0.10	-0.10	0.13	0.56	-0.37	1.00	-0.60	-0.70	0.12	0.34	0.52
InvSimpson	-0.60	-0.24	-0.35	-0.58	-0.10	0.05	0.06	-0.04	0.31	-0.47	0.15	-0.12	-0.52	0.65	-0.60	1.00	0.64	0.36	-0.01	-0.75
Firmicutes	-0.39	0.06	-0.09	-0.19	0.02	0.15	0.06	0.03	0.11	-0.15	0.27	-0.28	-0.26	0.28	-0.70	0.64	1.00	-0.29	-0.47	-0.68
Bacteroidota	0.05	-0.21	-0.18	-0.62	-0.31	-0.17	0.46	-0.45	-0.03	-0.27	-0.16	0.15	-0.28	0.60	0.12	0.36	-0.29	1.00	0.72	-0.46
B/F Ratio	0.42	0.02	0.01	-0.35	0.05	0.04	0.50	-0.23	0.10	0.00	-0.30	-0.02	-0.15	0.40	0.34	-0.01	-0.47	0.72	1.00	-0.13
Actinobacteriota	0.20	0.11	0.22	0.67	0.23	-0.05	-0.45	0.38	0.04	0.22	-0.05	0.18	0.47	-0.75	0.52	-0.75	-0.68	-0.46	-0.13	1.00

p-value	Glucose	Insulin	C-Peptide	HbA1c	Cholesterol	Triglyceride	HDL	LDL	Bodyfat	BCM	Adiponectin	E	CAP	Shannon	Chao1	InvSimpson	Firmicutes	Bacteroidota	B/F Ratio	Actinobacter iota
Glucose	NA	0.40	0.33	0.60	0.98	0.97	0.48	0.55	0.04	0.01	0.07	0.42	0.30	0.84	0.27	0.04	0.21	0.87	0.18	0.54
Insulin	0.40	NA	0.00	0.41	0.52	0.09	0.55	0.53	0.77	0.18	0.14	0.12	0.54	0.15	0.74	0.46	0.85	0.52	0.96	0.74
C-Peptide	0.33	0.00	NA	0.31	0.33	0.12	0.44	0.74	0.61	0.11	0.28	0.21	0.30	0.06	0.55	0.26	0.77	0.58	0.99	0.50
HbA1c	0.60	0.41	0.31	NA	0.33	0.87	0.42	0.23	0.48	0.31	0.45	0.26	0.30	0.00	0.71	0.05	0.55	0.03	0.27	0.02
Cholesterol	0.98	0.52	0.33	0.33	NA	0.35	0.23	0.00	0.26	0.56	0.51	0.64	0.16	0.16	0.69	0.77	0.94	0.33	0.89	0.48
Triglyceride	0.97	0.09	0.12	0.87	0.35	NA	0.90	0.50	0.83	0.31	0.81	0.06	0.23	0.94	0.65	0.89	0.65	0.60	0.89	0.88
HDL	0.48	0.55	0.44	0.42	0.23	0.90	NA	0.93	0.78	0.84	0.77	0.68	0.40	0.75	0.46	0.85	0.85	0.13	0.10	0.14
LDL	0.55	0.53	0.74	0.23	0.00	0.50	0.93	NA	0.22	0.97	0.44	0.75	0.10	0.18	0.93	0.90	0.93	0.14	0.47	0.22
Bodyfat	0.04	0.77	0.61	0.48	0.26	0.83	0.78	0.22	NA	0.03	0.10	0.89	0.62	0.74	0.94	0.32	0.73	0.92	0.75	0.91
BCM	0.01	0.18	0.11	0.31	0.56	0.31	0.84	0.97	0.03	NA	0.20	0.84	0.65	0.55	0.75	0.12	0.64	0.40	1.00	0.49
Adiponectin	0.07	0.14	0.28	0.45	0.51	0.81	0.77	0.44	0.10	0.20	NA	0.29	0.51	0.38	0.75	0.65	0.39	0.61	0.35	0.89
E	0.42	0.12	0.21	0.26	0.64	0.06	0.68	0.75	0.89	0.84	0.29	NA	0.66	0.93	0.69	0.72	0.38	0.65	0.96	0.59
CAP	0.30	0.54	0.30	0.30	0.16	0.23	0.40	0.10	0.62	0.65	0.51	0.66	NA	0.02	0.06	0.09	0.42	0.37	0.64	0.12
Shannon	0.84	0.15	0.06	0.00	0.16	0.94	0.75	0.18	0.74	0.55	0.38	0.93	0.02	NA	0.24	0.02	0.38	0.04	0.19	0.00
Chao1	0.27	0.74	0.55	0.71	0.69	0.65	0.46	0.93	0.94	0.75	0.75	0.69	0.06	0.24	NA	0.04	0.01	0.71	0.28	0.08
InvSimpson	0.04	0.46	0.26	0.05	0.77	0.89	0.85	0.90	0.32	0.12	0.65	0.72	0.09	0.02	0.04	NA	0.03	0.25	0.98	0.01
Firmicutes	0.21	0.85	0.77	0.55	0.94	0.65	0.85	0.93	0.73	0.64	0.39	0.38	0.42	0.38	0.01	0.03	NA	0.36	0.12	0.01
Bacteroidota	0.87	0.52	0.58	0.03	0.33	0.60	0.13	0.14	0.92	0.40	0.61	0.65	0.37	0.04	0.71	0.25	0.36	NA	0.01	0.14
B/F Ratio	0.18	0.96	0.99	0.27	0.89	0.89	0.10	0.47	0.75	1.00	0.35	0.96	0.64	0.19	0.28	0.98	0.12	0.01	NA	0.69
Actinobacteriota	0.54	0.74	0.50	0.02	0.48	0.88	0.14	0.22	0.91	0.49	0.89	0.59	0.12	0.00	0.08	0.01	0.01	0.14	0.69	NA

Supplementary Table S3: Correlation Matrix Pilsener. Data for Figure 5B. Correlation of delta for different variables for subjects consuming pilsener. First Table shows the correlation coefficient and the second table the p-value. Correlation was calculated with Pearson. B/F Ratio: Bacteroides-Firmicutes-Ratio, BCM: Body cell mass, CAP: Controlled attenuation parameter, E: Liver stiffness, HDL: high density lipoprotein, LDL: low density lipoprotein

R^2	Glucose	Insulin	C-Peptide	HbA1c	Cholesterol	Triglyceride	HDL	LDL	Bodyfat	BCM	Adiponectin	E	CAP	Shannon	Chao1	InvSimpson	Firmicutes	Bacteroidota	B/F Ratio	Actinobacteriota
Glucose	1.00	-0.44	-0.58	-0.34	-0.28	0.04	-0.59	-0.14	-0.56	0.74	-0.42	0.59	-0.24	0.22	0.00	0.31	-0.14	0.18	0.16	0.03
Insulin	-0.44	1.00	0.52	-0.19	-0.05	-0.32	0.51	-0.16	0.43	-0.28	0.77	0.24	-0.39	-0.55	-0.06	-0.65	-0.49	-0.09	-0.02	0.56
C-Peptide	-0.58	0.52	1.00	-0.35	0.06	-0.11	0.47	-0.02	-0.12	-0.58	0.48	-0.25	0.16	-0.06	0.01	-0.20	-0.45	0.36	0.13	0.16
HbA1c	-0.34	-0.19	-0.35	1.00	0.42	0.06	0.30	0.43	0.36	0.07	-0.35	-0.14	-0.20	0.33	0.36	0.38	0.42	-0.24	0.24	-0.26
Cholesterol	-0.28	-0.05	0.06	0.42	1.00	0.73	0.29	0.86	-0.10	-0.06	-0.08	-0.24	0.15	0.09	0.19	0.17	-0.26	0.13	0.37	0.21
Triglyceride	0.04	-0.32	-0.11	0.06	0.73	1.00	-0.17	0.60	-0.18	-0.22	-0.22	-0.19	0.49	0.06	-0.03	0.03	-0.19	0.17	0.27	0.12
HDL	-0.59	0.51	0.47	0.30	0.29	-0.17	1.00	-0.03	0.30	-0.30	0.08	-0.09	-0.27	0.03	0.23	-0.20	-0.24	0.38	0.34	-0.13
LDL	-0.14	-0.16	-0.02	0.43	0.86	0.60	-0.03	1.00	-0.23	0.18	-0.10	-0.12	0.09	0.18	0.34	0.34	-0.25	0.01	0.33	0.31
Bodyfat	-0.56	0.43	-0.12	0.36	-0.10	-0.18	0.30	-0.23	1.00	-0.41	0.34	-0.14	-0.04	-0.40	-0.12	-0.50	0.46	-0.52	-0.42	-0.11
BCM	0.74	-0.28	-0.58	0.07	-0.06	-0.22	-0.30	0.18	-0.41	1.00	-0.33	0.59	-0.58	0.25	0.24	0.46	-0.10	0.07	0.18	0.07
Adiponectin	-0.42	0.77	0.48	-0.35	-0.08	-0.22	0.08	-0.10	0.34	-0.33	1.00	0.02	-0.16	-0.75	-0.54	-0.59	-0.26	-0.35	-0.36	0.61
E	0.59	0.24	-0.25	-0.14	-0.24	-0.19	-0.09	-0.12	-0.14	0.59	0.02	1.00	-0.80	0.13	0.09	0.11	-0.43	0.32	0.50	0.20
CAP	-0.24	-0.39	0.16	-0.20	0.15	0.49	-0.27	0.09	-0.04	-0.58	-0.16	-0.80	1.00	-0.02	-0.04	-0.11	0.29	-0.16	-0.43	-0.18
Shannon	0.22	-0.55	-0.06	0.33	0.09	0.06	0.03	0.18	-0.40	0.25	-0.75	0.13	-0.02	1.00	0.62	0.86	0.15	0.52	0.55	-0.63
Chao1	0.00	-0.06	0.01	0.36	0.19	-0.03	0.23	0.34	-0.12	0.24	-0.54	0.09	-0.04	0.62	1.00	0.36	-0.15	0.23	0.39	-0.12
InvSimpson	0.31	-0.65	-0.20	0.38	0.17	0.03	-0.20	0.34	-0.50	0.46	-0.59	0.11	-0.11	0.86	0.36	1.00	0.25	0.26	0.39	-0.46
Firmicutes	-0.14	-0.49	-0.45	0.42	-0.26	-0.19	-0.24	-0.25	0.46	-0.10	-0.26	-0.43	0.29	0.15	-0.15	0.25	1.00	-0.53	-0.56	-0.65
Bacteroidota	0.18	-0.09	0.36	-0.24	0.13	0.17	0.38	0.01	-0.52	0.07	-0.35	0.32	-0.16	0.52	0.23	0.26	-0.53	1.00	0.74	-0.30
B/F Ratio	0.16	-0.02	0.13	0.24	0.37	0.27	0.34	0.33	-0.42	0.18	-0.36	0.50	-0.43	0.55	0.39	0.39	-0.56	0.74	1.00	-0.01
Actinobacteriota	0.03	0.56	0.16	-0.26	0.21	0.12	-0.13	0.31	-0.11	0.07	0.61	0.20	-0.18	-0.63	-0.12	-0.46	-0.65	-0.30	-0.01	1.00

p-value	Glucose	Insulin	C-Peptide	HbA1c	Cholesterol	Triglyceride	HDL	LDL	Bodyfat	BCM	Adiponectin	E	CAP	Shannon	Chao1	InvSimpson	Firmicutes	Bacteroidota	B/F Ratio	Actinobacteriota
Glucose	NA	0.17	0.06	0.31	0.41	0.91	0.06	0.67	0.07	0.01	0.20	0.06	0.48	0.52	0.99	0.36	0.68	0.60	0.64	0.93
Insulin	0.17	NA	0.10	0.57	0.88	0.34	0.11	0.65	0.19	0.41	0.01	0.48	0.23	0.08	0.87	0.03	0.13	0.80	0.95	0.07
C-Peptide	0.06	0.10	NA	0.29	0.85	0.75	0.14	0.94	0.73	0.06	0.13	0.46	0.64	0.86	0.97	0.55	0.16	0.28	0.71	0.63
HbA1c	0.31	0.57	0.29	NA	0.20	0.87	0.38	0.19	0.28	0.83	0.29	0.69	0.56	0.32	0.28	0.24	0.20	0.49	0.47	0.44
Cholesterol	0.41	0.88	0.85	0.20	NA	0.01	0.40	0.00	0.77	0.86	0.81	0.48	0.66	0.79	0.59	0.61	0.44	0.70	0.27	0.54
Triglyceride	0.91	0.34	0.75	0.87	0.01	NA	0.62	0.05	0.60	0.52	0.52	0.58	0.13	0.87	0.94	0.93	0.57	0.62	0.42	0.74
HDL	0.06	0.11	0.14	0.38	0.40	0.62	NA	0.92	0.38	0.36	0.81	0.80	0.42	0.92	0.50	0.57	0.48	0.25	0.30	0.70
LDL	0.67	0.65	0.94	0.19	0.00	0.05	0.92	NA	0.49	0.60	0.77	0.72	0.80	0.60	0.31	0.31	0.46	0.97	0.32	0.36
Bodyfat	0.07	0.19	0.73	0.28	0.77	0.60	0.38	0.49	NA	0.22	0.30	0.69	0.90	0.22	0.72	0.12	0.16	0.10	0.20	0.75
BCM	0.01	0.41	0.06	0.83	0.86	0.52	0.36	0.60	0.22	NA	0.32	0.06	0.06	0.45	0.48	0.15	0.76	0.85	0.59	0.83
Adiponectin	0.20	0.01	0.13	0.29	0.81	0.52	0.81	0.77	0.30	0.32	NA	0.95	0.65	0.01	0.09	0.06	0.43	0.30	0.28	0.05
E	0.06	0.48	0.46	0.69	0.48	0.58	0.80	0.72	0.69	0.06	0.95	NA	0.00	0.71	0.80	0.75	0.19	0.34	0.12	0.56
CAP	0.48	0.23	0.64	0.56	0.66	0.13	0.42	0.80	0.90	0.06	0.65	0.00	NA	0.95	0.90	0.74	0.38	0.63	0.19	0.60
Shannon	0.52	0.08	0.86	0.32	0.79	0.87	0.92	0.60	0.22	0.45	0.01	0.71	0.95	NA	0.04	0.00	0.66	0.10	0.08	0.04
Chao1	0.99	0.87	0.97	0.28	0.59	0.94	0.50	0.31	0.72	0.48	0.09	0.80	0.90	0.04	NA	0.28	0.67	0.49	0.23	0.72
InvSimpson	0.36	0.03	0.55	0.24	0.61	0.93	0.57	0.31	0.12	0.15	0.06	0.75	0.74	0.00	0.28	NA	0.46	0.44	0.23	0.15
Firmicutes	0.68	0.13	0.16	0.20	0.44	0.57	0.48	0.46	0.16	0.76	0.43	0.19	0.38	0.66	0.67	0.46	NA	0.10	0.07	0.03
Bacteroidota	0.60	0.80	0.28	0.49	0.70	0.62	0.25	0.97	0.10	0.85	0.30	0.34	0.63	0.10	0.49	0.44	0.10	NA	0.01	0.38
B/F Ratio	0.64	0.95	0.71	0.47	0.27	0.42	0.30	0.32	0.20	0.59	0.28	0.12	0.19	0.08	0.23	0.23	0.07	0.01	NA	0.98
Actinobacteriota	0.93	0.07	0.63	0.44	0.54	0.74	0.70	0.36	0.75	0.83	0.05	0.56	0.60	0.04	0.72	0.15	0.03	0.38	0.98	NA

Supplementary Table S4: Correlation Matrix Wheat Beer. Data for Figure 5C. Correlation of delta for different variables for subjects consuming wheat beer. First Table shows the correlation coefficient and the second table the p-value. Correlation was

calculated with Pearson. B/F Ratio: Bacteroides-Firmicutes-Ratio, BCM: Body cell mass, CAP: Controlled attenuation parameter, E: Liver stiffness, HDL: high density lipoprotein, LDL: low density lipoprotein

R^2	Glucose	Insulin	C-Peptide	HbA1c	Cholesterol	Triglyceride	HDL	LDL	Bodyfat	BCM	Adiponectin	E	CAP	Shannon	Chao1	InvSimpson	Firmicutes	Bacteroidota	B/F Ratio	Actinobacter iota
Glucose	1.00	0.15	0.15	0.24	0.71	0.32	-0.45	0.71	-0.26	0.32	-0.32	-0.60	0.64	0.01	-0.04	-0.20	-0.35	0.42	0.33	0.02
Insulin	0.15	1.00	0.98	-0.27	0.18	0.37	-0.45	0.25	0.24	-0.37	0.67	-0.13	-0.33	0.01	0.00	-0.20	-0.27	0.03	0.41	0.22
C-Peptide	0.15	0.98	1.00	-0.37	0.23	0.51	-0.49	0.28	0.13	-0.29	0.64	-0.22	-0.38	0.09	0.06	-0.07	-0.27	0.07	0.48	0.19
HbA1c	0.24	-0.27	-0.37	1.00	-0.02	-0.63	0.19	0.06	0.54	-0.17	-0.09	-0.01	0.29	-0.39	-0.52	-0.35	-0.26	-0.03	-0.28	0.26
Cholesterol	0.71	0.18	0.23	-0.02	1.00	0.57	-0.34	0.96	-0.16	0.26	-0.43	-0.81	0.48	0.43	0.21	0.16	-0.03	-0.08	0.35	0.06
Triglyceride	0.32	0.37	0.51	-0.63	0.57	1.00	-0.65	0.58	-0.42	0.42	-0.09	-0.65	-0.04	0.42	0.18	0.37	-0.26	0.18	0.70	0.10
HDL	-0.45	-0.45	-0.49	0.19	-0.34	-0.65	1.00	-0.49	0.09	-0.34	-0.13	0.31	-0.12	0.25	0.45	0.34	0.72	-0.30	-0.63	-0.46
LDL	0.71	0.25	0.28	0.06	0.96	0.58	-0.49	1.00	-0.05	0.28	-0.35	-0.85	0.45	0.31	-0.03	0.03	-0.25	-0.12	0.42	0.29
Bodyfat	-0.26	0.24	0.13	0.54	-0.16	-0.42	0.09	-0.05	1.00	-0.56	0.46	0.15	-0.17	-0.18	-0.32	-0.13	0.00	-0.19	0.05	0.12
BCM	0.32	-0.37	-0.29	-0.17	0.26	0.42	-0.34	0.28	-0.56	1.00	-0.62	-0.42	0.61	0.30	-0.17	0.25	-0.33	0.41	0.38	0.03
Adiponectin	-0.32	0.67	0.64	-0.09	-0.43	-0.09	-0.13	-0.35	0.46	-0.62	1.00	0.34	-0.69	-0.44	-0.24	-0.20	-0.07	0.01	0.00	0.06
E	-0.60	-0.13	-0.22	-0.01	-0.81	-0.65	0.31	-0.85	0.15	-0.42	0.34	1.00	-0.30	-0.50	-0.03	-0.43	0.22	-0.05	-0.51	-0.15
CAP	0.64	-0.33	-0.38	0.29	0.48	-0.04	-0.12	0.45	-0.17	0.61	-0.69	-0.30	1.00	0.21	-0.01	-0.09	-0.07	0.35	0.18	-0.18
Shannon	0.01	0.01	0.09	-0.39	0.43	0.42	0.25	0.31	-0.18	0.30	-0.44	-0.50	0.21	1.00	0.68	0.76	0.35	0.01	0.43	-0.34
Chao1	-0.04	0.00	0.06	-0.52	0.21	0.18	0.45	-0.03	-0.32	-0.17	-0.24	-0.03	-0.01	0.68	1.00	0.51	0.71	0.01	0.04	-0.66
InvSimpson	-0.20	-0.20	-0.07	-0.35	0.16	0.37	0.34	0.03	-0.13	0.25	-0.20	-0.43	-0.09	0.76	0.51	1.00	0.46	0.15	0.31	-0.53
Firmicutes	-0.35	-0.27	-0.27	-0.26	-0.03	-0.26	0.72	-0.25	0.00	-0.33	-0.07	0.22	-0.07	0.35	0.71	0.46	1.00	-0.24	-0.41	-0.75
Bacteroidota	0.42	0.03	0.07	-0.03	-0.08	0.18	-0.30	-0.12	-0.19	0.41	0.01	-0.05	0.35	0.01	0.01	0.15	-0.24	1.00	0.54	-0.46
B/F Ratio	0.33	0.41	0.48	-0.28	0.35	0.70	-0.63	0.42	0.05	0.38	0.00	-0.51	0.18	0.43	0.04	0.31	-0.41	0.54	1.00	0.01
Actinobacteriota	0.02	0.22	0.19	0.26	0.06	0.10	-0.46	0.29	0.12	0.03	0.06	-0.15	-0.18	-0.34	-0.66	-0.53	-0.75	-0.46	0.01	1.00

p-value	Glucose	Insulin	C-Peptide	HbA1c	Cholesterol	Triglyceride	HDL	LDL	Bodyfat	BCM	Adiponectin	E	CAP	Shannon	Chao1	InvSimpson	Firmicutes	Bacteroidota	B/F Ratio	Actinobacter iota
Glucose	NA	0.65	0.65	0.48	0.01	0.34	0.17	0.01	0.45	0.34	0.34	0.05	0.03	0.99	0.91	0.55	0.29	0.20	0.32	0.96
Insulin	0.65	NA	0.00	0.43	0.59	0.27	0.16	0.45	0.47	0.26	0.02	0.71	0.32	0.98	0.99	0.55	0.41	0.94	0.21	0.51
C-Peptide	0.65	0.00	NA	0.26	0.50	0.11	0.13	0.41	0.69	0.38	0.03	0.52	0.25	0.80	0.87	0.84	0.42	0.84	0.13	0.58
HbA1c	0.48	0.43	0.26	NA	0.95	0.04	0.58	0.85	0.09	0.62	0.79	0.97	0.39	0.23	0.10	0.29	0.44	0.92	0.41	0.45
Cholesterol	0.01	0.59	0.50	0.95	NA	0.07	0.31	0.00	0.65	0.45	0.19	0.00	0.13	0.19	0.54	0.64	0.93	0.82	0.28	0.86
Triglyceride	0.34	0.27	0.11	0.04	0.07	NA	0.03	0.06	0.19	0.20	0.80	0.03	0.91	0.20	0.60	0.26	0.44	0.59	0.02	0.76
HDL	0.17	0.16	0.13	0.58	0.31	0.03	NA	0.13	0.78	0.31	0.69	0.36	0.73	0.47	0.16	0.31	0.01	0.38	0.04	0.16
LDL	0.01	0.45	0.41	0.85	0.00	0.06	0.13	NA	0.88	0.41	0.30	0.00	0.17	0.36	0.94	0.93	0.46	0.73	0.20	0.40
Bodyfat	0.45	0.47	0.69	0.09	0.65	0.19	0.78	0.88	NA	0.07	0.16	0.66	0.62	0.59	0.34	0.71	1.00	0.58	0.88	0.72
BCM	0.34	0.26	0.38	0.62	0.45	0.20	0.31	0.41	0.07	NA	0.04	0.20	0.05	0.37	0.63	0.46	0.31	0.21	0.25	0.93
Adiponectin	0.34	0.02	0.03	0.79	0.19	0.80	0.69	0.30	0.16	0.04	NA	0.30	0.02	0.17	0.48	0.55	0.84	0.97	1.00	0.87
E	0.05	0.71	0.52	0.97	0.00	0.03	0.36	0.00	0.66	0.20	0.30	NA	0.37	0.12	0.92	0.19	0.52	0.89	0.11	0.65
CAP	0.03	0.32	0.25	0.39	0.13	0.91	0.73	0.17	0.62	0.05	0.02	0.37	NA	0.53	0.97	0.80	0.83	0.30	0.60	0.60
Shannon	0.99	0.98	0.80	0.23	0.19	0.20	0.47	0.36	0.59	0.37	0.17	0.12	0.53	NA	0.02	0.01	0.29	0.97	0.19	0.31
Chao1	0.91	0.99	0.87	0.10	0.54	0.60	0.16	0.94	0.34	0.63	0.48	0.92	0.97	0.02	NA	0.11	0.01	0.97	0.91	0.03
InvSimpson	0.55	0.55	0.84	0.29	0.64	0.26	0.31	0.93	0.71	0.46	0.55	0.19	0.80	0.01	0.11	NA	0.15	0.66	0.35	0.10
Firmicutes	0.29	0.41	0.42	0.44	0.93	0.44	0.01	0.46	1.00	0.31	0.84	0.52	0.83	0.29	0.01	0.15	NA	0.47	0.21	0.01
Bacteroidota	0.20	0.94	0.84	0.92	0.82	0.59	0.38	0.73	0.58	0.21	0.97	0.89	0.30	0.97	0.97	0.66	0.47	NA	0.09	0.15
B/F Ratio	0.32	0.21	0.13	0.41	0.28	0.02	0.04	0.20	0.88	0.25	1.00	0.11	0.60	0.19	0.91	0.35	0.21	0.09	NA	0.99
Actinobacteriota	0.96	0.51	0.58	0.45	0.86	0.76	0.16	0.40	0.72	0.93	0.87	0.65	0.60	0.31	0.03	0.10	0.01	0.15	0.99	NA

Supplementary Table S5: Correlation Matrix Mixed Beer. Data for Figure 5D. Correlation of delta for different variables for subjects consuming mixed beer. First Table shows the correlation coefficient and the second table the p-value. Correlation was calculated with Pearson. B/F Ratio: Bacteroides-Firmicutes-Ratio, BCM: Body cell mass, CAP: Controlled attenuation parameter, E: Liver stiffness, HDL: high density lipoprotein, LDL: low density lipoprotein

R^2	Glucose	Insulin	C-Peptide	HbA1c	Cholesterol	Triglyceride	HDL	LDL	Bodyfat	BCM	Adiponectin	E	CAP	Shannon	Chao1	InvSimpson	Firmicutes	Bacteroidota	B/F Ratio	Actinobacter iota
Glucose	1.00	0.63	0.45	0.50	0.27	0.37	0.59	0.12	-0.33	0.49	0.21	-0.43	0.52	0.33	0.49	0.35	0.26	-0.18	-0.53	-0.21
Insulin	0.63	1.00	0.80	0.92	0.04	0.62	0.10	-0.10	-0.48	0.59	0.12	-0.67	0.07	0.37	0.54	0.47	-0.11	-0.02	-0.43	0.11
C-Peptide	0.45	0.80	1.00	0.74	-0.13	0.55	-0.02	-0.36	-0.54	0.46	-0.12	-0.60	0.42	-0.03	0.23	0.13	-0.39	-0.07	-0.28	0.59
HbA1c	0.50	0.92	0.74	1.00	0.12	0.67	0.08	0.04	-0.56	0.67	-0.03	-0.48	0.04	0.19	0.30	0.20	-0.21	0.02	-0.36	0.24
Cholesterol	0.27	0.04	-0.13	0.12	1.00	0.33	0.63	0.94	-0.37	0.27	-0.48	0.05	-0.17	0.62	0.44	0.32	0.48	-0.38	0.37	-0.19
Triglyceride	0.37	0.62	0.55	0.67	0.33	1.00	-0.05	0.20	-0.29	0.20	0.12	-0.17	-0.02	0.29	0.41	0.25	0.29	-0.64	-0.45	0.35
HDL	0.59	0.10	-0.02	0.08	0.63	-0.05	1.00	0.48	-0.48	0.50	-0.37	0.05	0.24	0.26	0.19	0.07	0.28	0.06	0.12	-0.48
LDL	0.12	-0.10	-0.36	0.04	0.94	0.20	0.48	1.00	-0.22	0.21	-0.40	0.13	-0.31	0.57	0.32	0.26	0.48	-0.31	0.38	-0.28
Bodyfat	-0.33	-0.48	-0.54	-0.56	-0.37	-0.29	-0.48	-0.22	1.00	-0.77	0.72	-0.03	-0.20	-0.04	-0.01	-0.09	-0.05	0.06	-0.05	-0.01
BCM	0.49	0.59	0.46	0.67	0.27	0.20	0.50	0.21	-0.77	1.00	-0.48	-0.40	0.27	0.08	-0.01	0.11	-0.01	0.07	-0.17	-0.08
Adiponectin	0.21	0.12	-0.12	-0.03	-0.48	0.12	-0.37	-0.40	0.72	-0.48	1.00	0.10	-0.09	0.04	0.24	0.14	0.14	-0.01	-0.64	-0.24
E	-0.43	-0.67	-0.60	-0.48	0.05	-0.17	0.05	0.13	-0.03	-0.40	0.10	1.00	-0.26	-0.29	-0.29	-0.44	0.11	0.14	0.16	-0.25
CAP	0.52	0.07	0.42	0.04	-0.17	-0.02	0.24	-0.31	-0.20	0.27	-0.09	-0.26	1.00	-0.45	-0.26	-0.30	-0.12	-0.16	-0.36	0.36
Shannon	0.33	0.37	-0.03	0.19	0.62	0.29	0.26	0.57	-0.04	0.08	0.04	-0.29	-0.45	1.00	0.92	0.91	0.47	-0.26	0.17	-0.39
Chao1	0.49	0.54	0.23	0.30	0.44	0.41	0.19	0.32	-0.01	-0.01	0.24	-0.29	-0.26	0.92	1.00	0.89	0.33	-0.25	-0.01	-0.24
InvSimpson	0.35	0.47	0.13	0.20	0.32	0.25	0.07	0.26	-0.09	0.11	0.14	-0.44	-0.30	0.91	0.89	1.00	0.44	-0.30	-0.03	-0.34
Firmicutes	0.26	-0.11	-0.39	-0.21	0.48	0.29	0.28	0.48	-0.05	-0.01	0.14	0.11	-0.12	0.47	0.33	0.44	1.00	-0.72	-0.32	-0.54
Bacteroidota	-0.18	-0.02	-0.07	0.02	-0.38	-0.64	0.06	-0.31	0.06	0.07	-0.01	0.14	-0.16	-0.26	-0.25	-0.30	-0.72	1.00	0.34	-0.20
B/F Ratio	-0.53	-0.43	-0.28	-0.36	0.37	-0.45	0.12	0.38	-0.05	-0.17	-0.64	0.16	-0.36	0.17	-0.01	-0.03	-0.32	0.34	1.00	0.07
Actinobacteriota	-0.21	0.11	0.59	0.24	-0.19	0.35	-0.48	-0.28	-0.01	-0.08	-0.24	-0.25	0.36	-0.39	-0.24	-0.34	-0.54	-0.20	0.07	1.00

p-value	Glucose	Insulin	C-Peptide	HbA1c	Cholesterol	Triglyceride	HDL	LDL	Bodyfat	BCM	Adiponectin	E	CAP	Shannon	Chao1	InvSimpson	Firmicutes	Bacteroidota	B/F Ratio	Actinobacter iota
Glucose	NA	0.07	0.23	0.17	0.48	0.32	0.10	0.76	0.39	0.18	0.58	0.25	0.15	0.39	0.18	0.36	0.50	0.64	0.15	0.59
Insulin	0.07	NA	0.01	0.00	0.92	0.08	0.79	0.80	0.19	0.09	0.75	0.05	0.85	0.33	0.14	0.21	0.77	0.97	0.25	0.77
C-Peptide	0.23	0.01	NA	0.02	0.74	0.13	0.96	0.34	0.13	0.22	0.76	0.09	0.26	0.95	0.55	0.74	0.30	0.87	0.47	0.10
HbA1c	0.17	0.00	0.02	NA	0.75	0.05	0.83	0.91	0.12	0.05	0.95	0.19	0.92	0.63	0.44	0.61	0.59	0.96	0.34	0.53
Cholesterol	0.48	0.92	0.74	0.75	NA	0.39	0.07	0.00	0.33	0.48	0.19	0.91	0.66	0.07	0.24	0.40	0.19	0.31	0.33	0.62
Triglyceride	0.32	0.08	0.13	0.05	0.39	NA	0.89	0.60	0.45	0.61	0.77	0.67	0.96	0.45	0.27	0.52	0.45	0.06	0.23	0.36
HDL	0.10	0.79	0.96	0.83	0.07	0.89	NA	0.19	0.20	0.17	0.33	0.90	0.53	0.49	0.63	0.86	0.46	0.88	0.75	0.19
LDL	0.76	0.80	0.34	0.91	0.00	0.60	0.19	NA	0.57	0.59	0.29	0.73	0.42	0.11	0.40	0.50	0.19	0.42	0.32	0.47
Bodyfat	0.39	0.19	0.13	0.12	0.33	0.45	0.20	0.57	NA	0.01	0.03	0.93	0.58	0.91	0.97	0.80	0.89	0.86	0.90	0.97
BCM	0.18	0.09	0.22	0.05	0.48	0.61	0.17	0.59	0.01	NA	0.19	0.25	0.45	0.83	0.99	0.76	0.98	0.84	0.64	0.83
Adiponectin	0.58	0.75	0.76	0.95	0.19	0.77	0.33	0.29	0.03	0.19	NA	0.80	0.83	0.93	0.54	0.72	0.71	0.98	0.06	0.53
E	0.25	0.05	0.09	0.19	0.91	0.67	0.90	0.73	0.93	0.25	0.80	NA	0.47	0.41	0.42	0.21	0.77	0.71	0.65	0.48
CAP	0.15	0.85	0.26	0.92	0.66	0.96	0.53	0.42	0.58	0.45	0.83	0.47	NA	0.19	0.47	0.40	0.73	0.65	0.31	0.30
Shannon	0.39	0.33	0.95	0.63	0.07	0.45	0.49	0.11	0.91	0.83	0.93	0.41	0.19	NA	0.00	0.00	0.18	0.46	0.64	0.26
Chao1	0.18	0.14	0.55	0.44	0.24	0.27	0.63	0.40	0.97	0.99	0.54	0.42	0.47	0.00	NA	0.00	0.35	0.48	0.99	0.51
InvSimpson	0.36	0.21	0.74	0.61	0.40	0.52	0.86	0.50	0.80	0.76	0.72	0.21	0.40	0.00	0.00	NA	0.20	0.40	0.93	0.33
Firmicutes	0.50	0.77	0.30	0.59	0.19	0.45	0.46	0.19	0.89	0.98	0.71	0.77	0.73	0.18	0.35	0.20	NA	0.02	0.37	0.11
Bacteroidota	0.64	0.97	0.87	0.96	0.31	0.06	0.88	0.42	0.86	0.84	0.98	0.71	0.65	0.46	0.48	0.40	0.02	NA	0.34	0.59
B/F Ratio	0.15	0.25	0.47	0.34	0.33	0.23	0.75	0.32	0.90	0.64	0.06	0.65	0.31	0.64	0.99	0.93	0.37	0.34	NA	0.85
Actinobacteriota	0.59	0.77	0.10	0.53	0.62	0.36	0.19	0.47	0.97	0.83	0.53	0.48	0.30	0.26	0.51	0.33	0.11	0.59	0.85	NA

Supplementary Table S6: Alpha diversity as calculated by Shannon, Chao1 and Inverse Simpson Index at week 0 and week 4 for all groups. Data is presented as median with minimal and maximal value. p-value was calculated with Wilcoxon signed rank test on paired samples.

ALPHA DIVERSITY	Week 0	Week 4	p-value
**Shannon-Index**			
Water	3.52 (2.77 – 4.33)	3.6 (2.14 – 4.11)	0.176
Pilsener	3.92 (3 – 4.52)	3.42 (2.78 – 4.25)	*<0.001*
Wheat Beer	3.8 (2.79 – 4.25)	3.6 (3.35 – 4.06)	0.898
Mixed Beer	3.66 (3.04 – 4.7)	3.76 (3.13 – 4.22)	0.846
**Chao1**			
Water	230 (132 – 417)	187 (71 – 395)	*0.00418*
Pilsener	251 (125 – 416)	185 (113 – 371)	*0.0186*
Wheat Beer	237 (109 – 426)	195 (151 – 364)	0.175
Mixed Beer	206 (110 – 486)	236 (110 – 324)	0.906
**Inverse Simpson**			
Water	15 (5.57 – 42.6)	20.7 (4.97 – 30.3)	0.569
Pilsener	28.7 (12-5 – 39.9)	14.9 (6.32 – 37.6)	*0.00195*
Wheat Beer	20.1 (7.04 – 36.7)	20.3 (13.7 - 30.7)	0.898
Mixed Beer	19.5 (6.27 – 59.7)	21 (12.4 – 40.1)	0.625

Supplementary Table S7: Relative Abundance for selected bacteria at week 0 and week 4 for all groups. Data is presented as median and minimal/maximal values. Statistically significance was calculated with Wilcoxon signed rank test on paired samples.

RELATIVE ABUNDANCE	Week 0	Week 4	p-value
**Firmicutes**			
Water	0.708 (0.281 – 0.943	0.705 (0.506 – 0.984)	0.39
Pilsener	0.705 (0.592 – 0.869)	0.637 (0.455 – 0.806)	*<0.001*
Wheat Beer	0.766 (0.418 – 0.92)	0.725 (0.427 – 0.895)	0.898
Mixed Beer	0.817 (0.391 – 0.944)	0.81 (0.583 – 0.921)	0.105
**Actinobacteriota**			
Water	0.191 (0.019 – 0.627)	0.156 (0.007 – 0.454)	0.339
Pilsener	0.158 (0.037 – 0.331)	0.18 (0.021 – 0.518)	*0.002*
Wheat Beer	0.087 (0.03 – 0.423)	0.196 (0.079 – 0.304)	0.067
Mixed Beer	0.08 (0.017 – 0.222)	0.075 (0.019 – 0.367)	1
**Bifidobacterium**			
Water	0.096 (0.009 – 0.4)	0.075 (0 – 0.339)	0.11
Pilsener	0.053 (0.008 – 0.221)	0.117 (0.01 – 0.427)	*<0.001*
Wheat Beer	0.047 (0.002 – 0.277)	0.114 (0.028 – 0.187)	0.054
Mixed Beer	0.051 (0.002 – 0.188)	0.027 (0.004 – 0.229)	0.322
**Bacteroides**			
Water	0.036 (0.002 – 0.147)	0.006 (0 – 0.264)	*0.034*
Pilsener	0.03 (0.002 – 0.193)	0.018 (0.003 – 0.182)	*0.024*
Wheat Beer	0.026 (0.004 – 0.101)	0.016 (0.001 – 0.173)	0.054
Mixed Beer	0.057 (0.002 – 0.271)	0.046 (0 – 0.069)	*0.002*

Supplementary Figures

Supplementary Figure S1

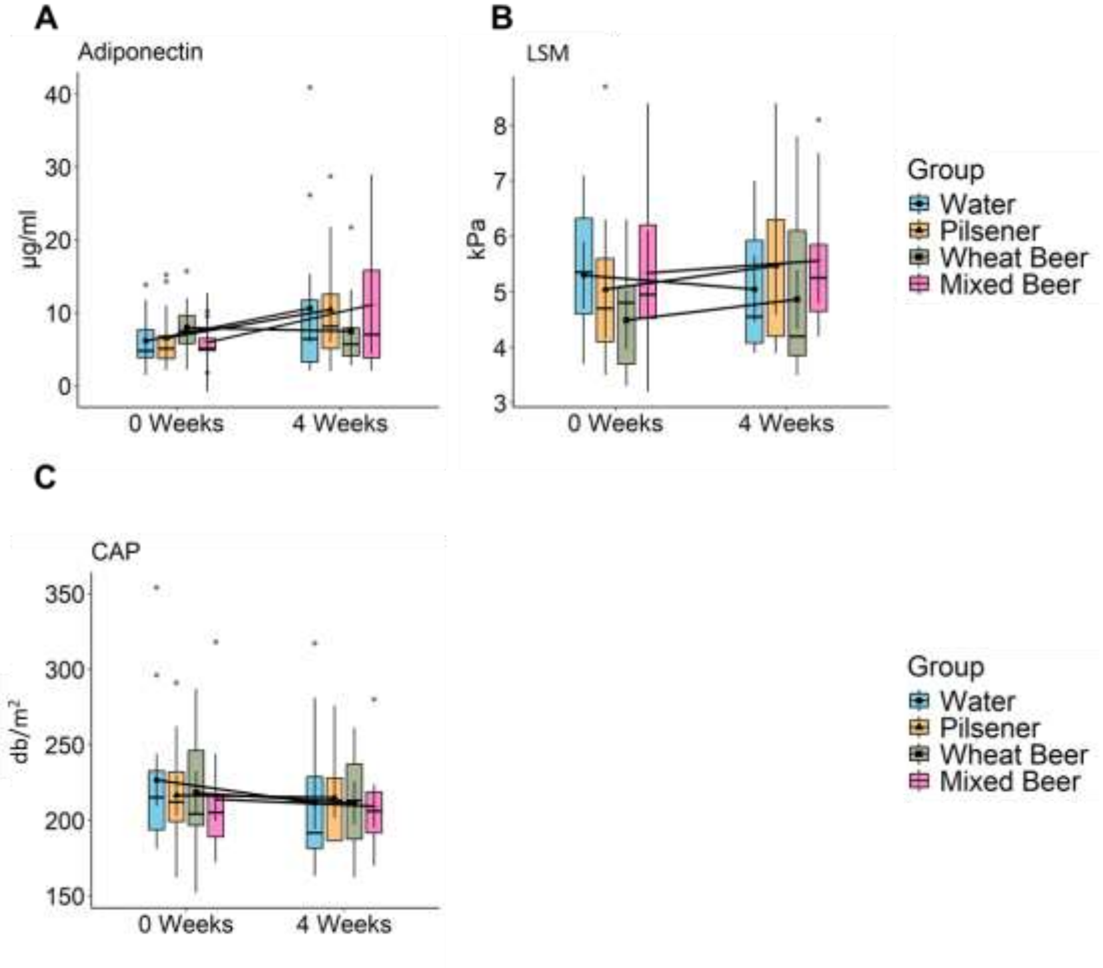

Supplementary Figure S1: Non-alcoholic beer consumption did not alter liver stiffness or liver steatosis as measured by transient elastography. Adiponectin-levels (A), liver stiffness/LSM (B) and liver steatosis/CAP (C) were measured in healthy young men at baseline and after 4 weeks consumption of 660ml daily of either pilsener (PI; n=11), wheat beer (WB; n=11) or mixed beer (MB; n=10). Water consumption (WA; n=12) was included as a control group. Estimated marginal means and error bars (95% confidence interval) in the foreground and raw data is plotted in the background. Significance was calculated using two way repeated measures ANOVA. A Adiponectin (WA: p=0.28, PI: p=0.49, WB: p=0.51, MB: p=0.65), B LSM (WA: p=0.58, PI: p=0.36, WB: p=0.014, MB: p=0.36), C CAP (WA: p=0.68, PI: p=0.81, WB: p=0.134, MB: p=0.14).

Supplementary Figure S2

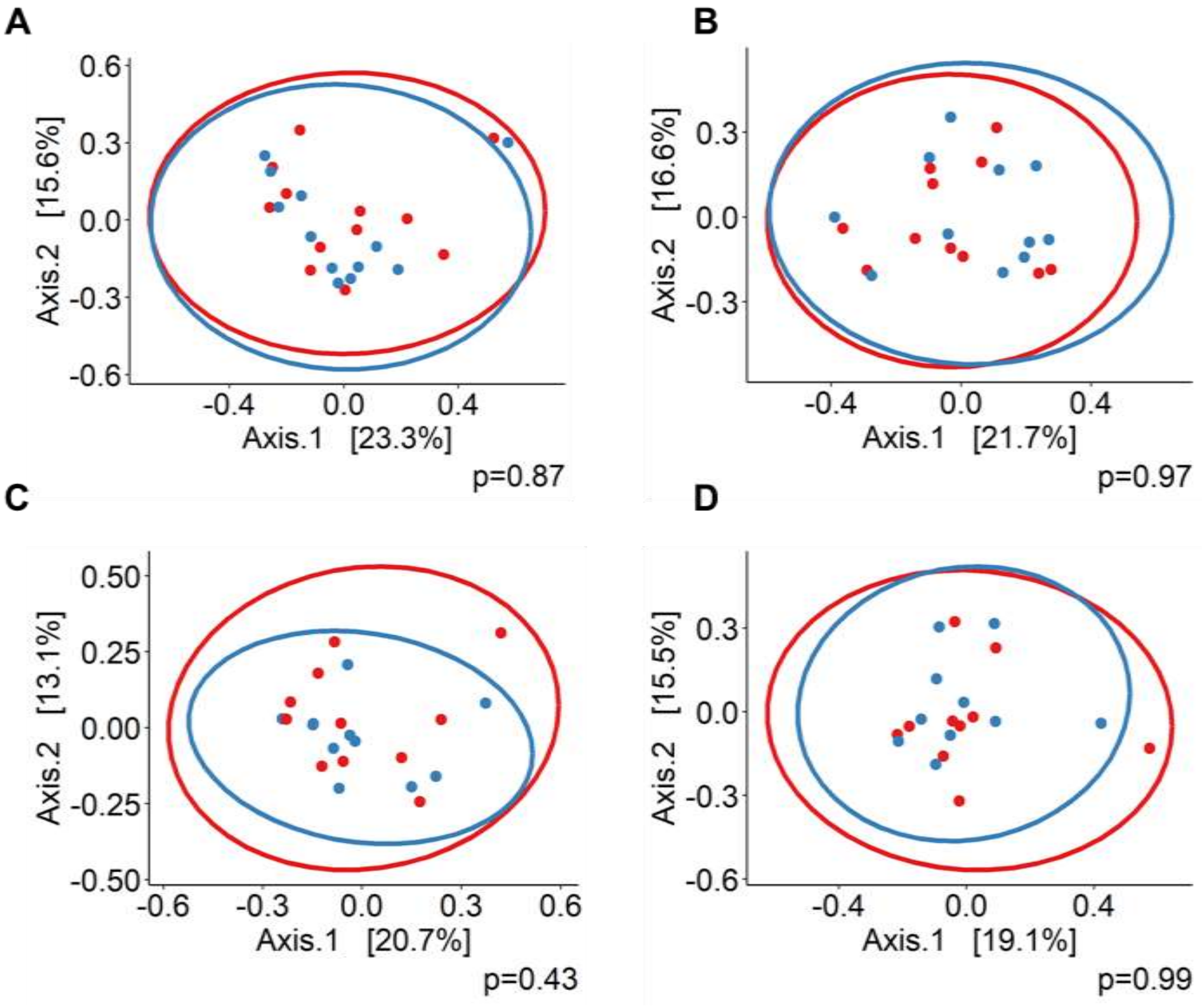

Supplementary Figure S2: Beta diversity did not change after 4 week consumption of non-alcoholic beers. Beta Diversity was calculated by principal cluster analysis using bray-curtis distance model. Significance was calculated with Permutational Multivariate Analysis of Variance Using Distance Matrices (PERMANOVA). A: Water, B: Pilsener, C: Wheat Beer, D: Mixed Beer.

Supplementary Figure S3

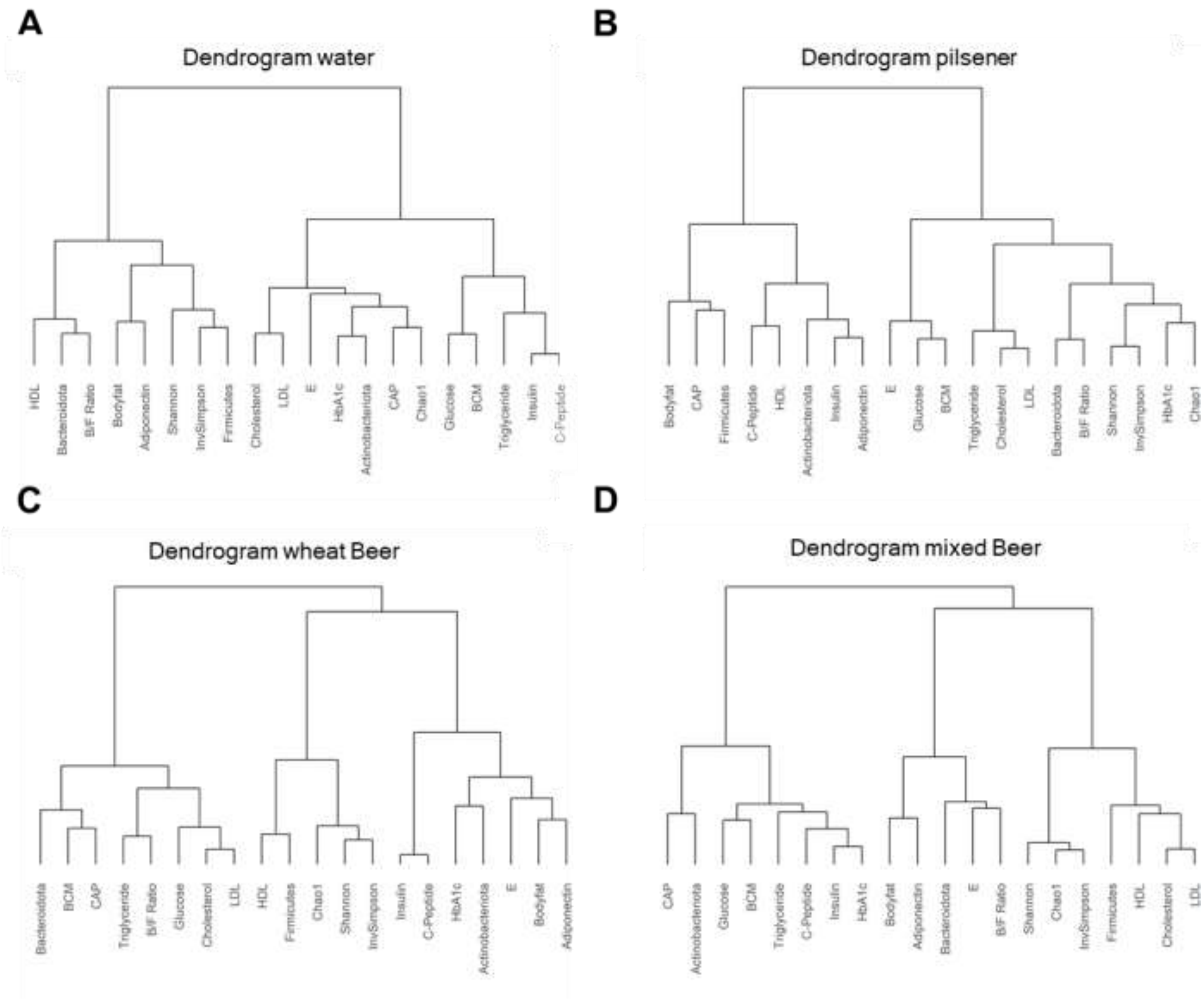

Supplementary Figure S3: Dendrogram for hierarchical clustering in correlation matrix from figure 5. A: Water, B: Pilsener, C: Mixed Beer, D: Wheat Beer.
